# Supplementary material for: Imaging mitochondrial membrane potential via concentration-dependent fluorescence lifetime changes
Source: Nat Commun. 2025 Dec 12;16:11088. doi: 10.1038/s41467-025-66042-x (PMC12700901; doi:10.1038/s41467-025-66042-x)
Supplement: Supplementary file 1 — Supplementary Information [file 41467_2025_66042_MOESM1_ESM.pdf]

Supplementary Information

# Imaging mitochondrial membrane potential via concentration-dependent fluorescence lifetime changes

Dilizhatai·Saimi[1], Luc Reymond[2], Tursunjan Aziz[3], Xuan Shen[4], Ziying Luo[1,5], Shuaibo Pi[1], Yitong Liu[1,5], Song Fu[6,7,8], Shuangjin Ding[1], Anming Meng[3,9,10], Liangyi Chen[1,11,12], Hui Jiang[7,8,13], Zhixing Chen[1,5,11,12,14]

1. College of Future Technology, Institute of Molecular Medicine, National Biomedical Imaging Center, Beijing Key Laboratory of Cardiometabolic Molecular Medicine, Peking University, Beijing 100871, China
2. Biomolecular Screening Facility, École Polytechnique Fédérale de Lausanne (EPFL), Lausanne 1015, Switzerland
3. Laboratory of Molecular Developmental Biology, State Key Laboratory of Membrane Biology, Tsinghua-Peking Center for Life Sciences, School of Life Sciences, Tsinghua University, Beijing 100084, China
4. College of Chemistry and Molecular Engineering, Synthetic and Functional Biomolecules Center, Beijing National Laboratory for Molecular Sciences, Key Laboratory of Bioorganic Chemistry and Molecular Engineering of Ministry of Education, Peking University, Beijing, 100871 China
5. Peking-Tsinghua Center for Life Science, Academy for Advanced Interdisciplinary Studies, Peking University, Beijing 100871, China
6. Graduate School of Peking Union Medical College, Beijing 100730, China
7. National Institute of Biological Sciences, Beijing 102206, China
8. Beijing Key Laboratory of Cell Biology for Animal Aging, Beijing 102206, China
9. Developmental Diseases and Cancer Research Center, Sun Yat-sen Memorial Hospital, Sun Yat-sen University, Guangzhou 510120, China
10. Laboratory of Stem Cell Regulation, Guangzhou Laboratory, Guangzhou 510320, China
11. PKU-Nanjing Institute of Translational Medicine, Nanjing 211800, China
12. State Key Laboratory of Membrane Biology, Peking University, Beijing, China
13. Tsinghua Institute of Multidisciplinary Biomedical Research, Tsinghua University, Beijing 102206, China
14. GenVivo Tech, Nanjing 211800, China

*Corresponding author: Zhixing Chen (zhixingchen@pku.edu.cn)

**This PDF file includes:**

Supplementary information text:

Supplementary Figures 1-11

Supplementary Table 1-2

Synthesis and characterization of compound

Supplementary References

**Other supplementary materials for this manuscript include the following:**

Supplementary Movies 1-3

# Supplementary Figures

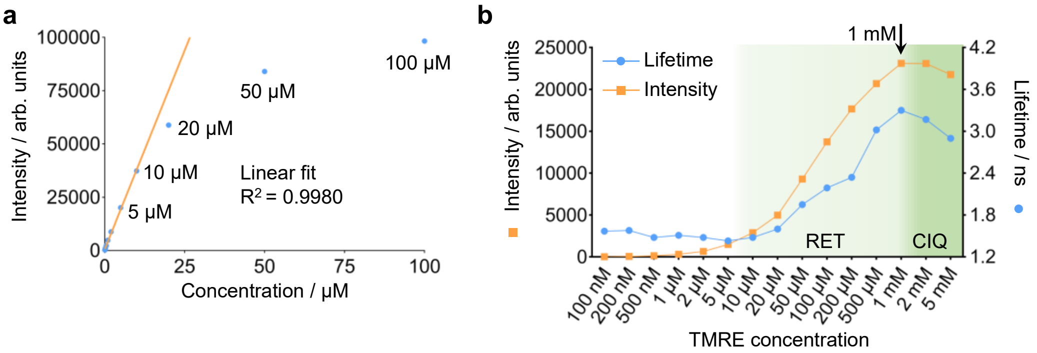


**Supplementary Figure 1.** Dyes at high concentrations show decreased apparent fluorescence lifetimes. **(a)** Fluorescence intensity (blue dot) of PKMDR in DMSO at various concentrations exhibits a linear relationship at lower concentrations (orange line) and a saturation plateau at higher concentrations. **(b)** Co-plot of fluorescence intensity (orange dot) and fluorescence lifetime (blue dot) of TMRE in DMSO at various concentrations showing an aggregation-induced fluorescence lifetime decrease. Abbreviations: RET, radiative energy transport; CIQ, concentration-induced quenching.

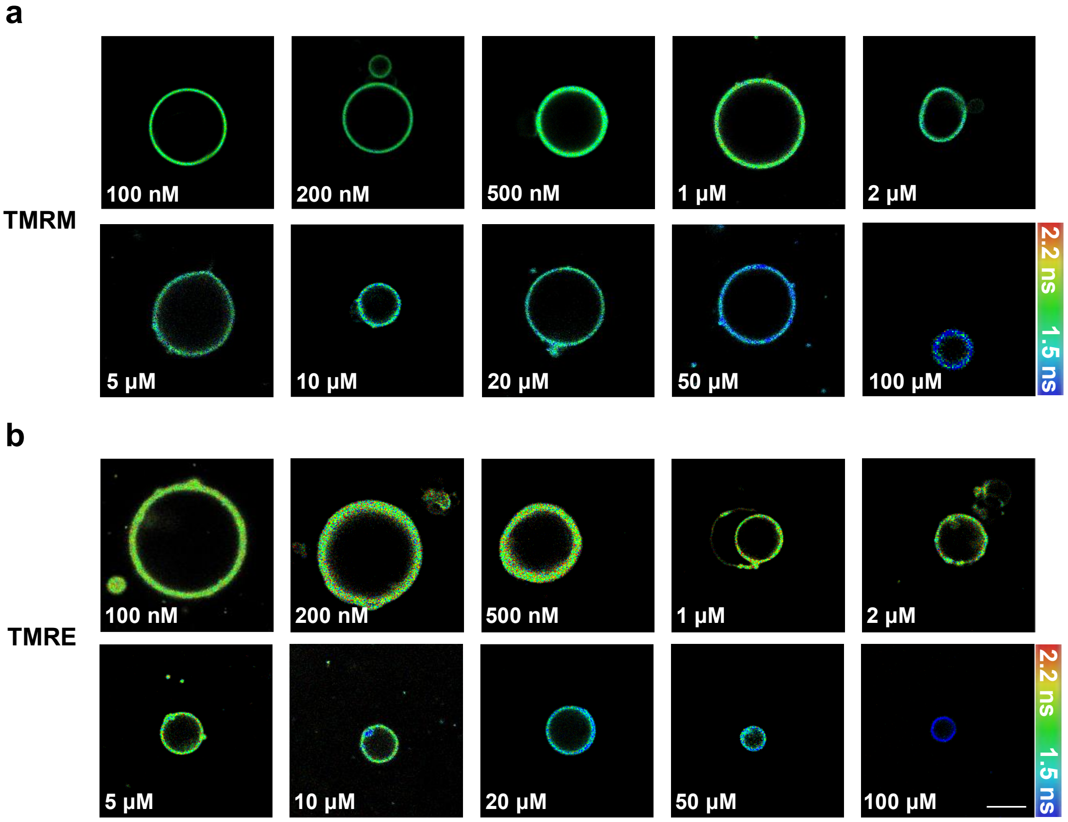


**Supplementary Figure 2.** Fluorescence lifetime imaging of TMRM **(a)** and TMRE **(b)** in 1, 2 dioleoyl sn-glycero-3-phosphocholine (DOPC) giant unilamellar vesicles (GUVs) at various concentrations. Scale bar, 10 μm.

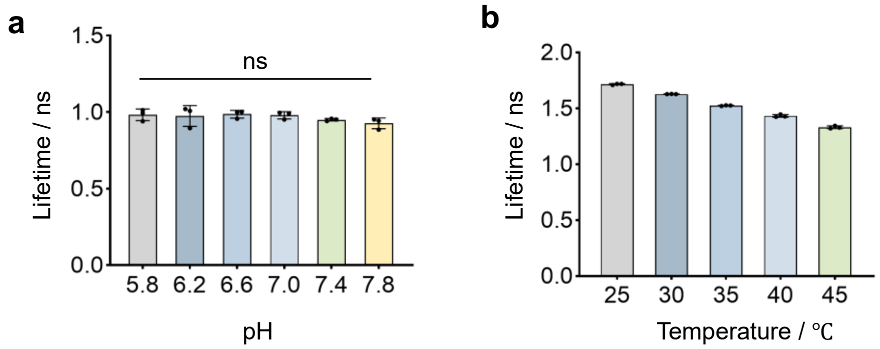


**Supplementary Figure 3.** Fluorescence lifetime plot of PKMDR at various pH (a) and temperature (b). Data were presented as the mean ± SEM, n=3 samples.

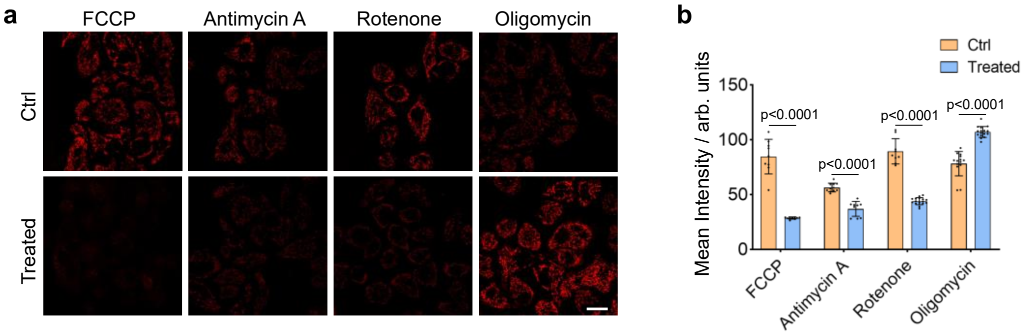


**Supplementary Figure 4**. Observe the change of membrane potential using TMRM. **(a)**, Confocal image of HeLa cells treated with FCCP (OXPHOS uncouplers), antimycin A (complex Ⅲ inhibitor), rotenone (complex I inhibitor) and oligomycin (ATP synthase inhibitor). Scale bar, 20 μM. **(b)**, Corresponding bar plots showing the average fluorescence intensity change of mitochondria treated with FCCP (n = 9 cells), antimycin A (n = 11 cells), rotenone (n = 9 cells) or oligomycin (n = 14 cells). Data were presented as the mean ± SEM. P-values were calculated using unpaired two-tailed Student's t-test.

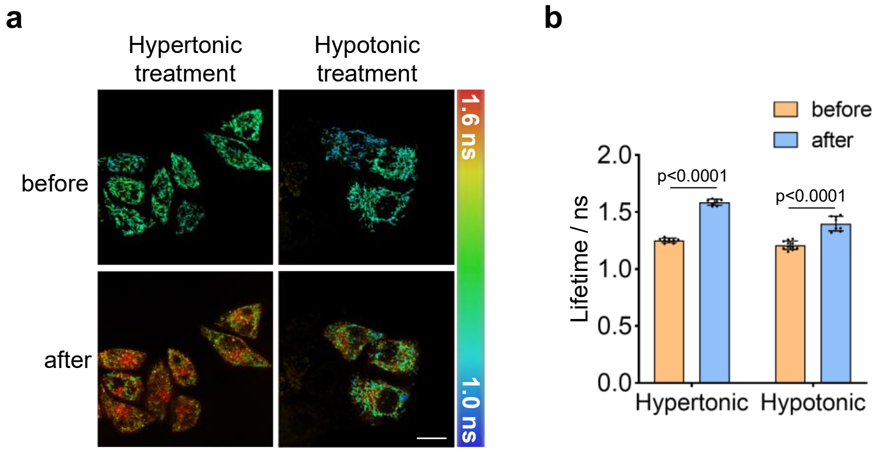


**Supplementary Figure 5**. PKMDR does not respond to changes in membrane tension. **(a)** FLIM image of HeLa cell before and after hypertonic and hypotonic treatment. Scale bar = 20 µm. **(b)** Plots showing the average fluorescence lifetime observed with hypertonic (n = 8 cells) and hypotonic treatment (n = 11 cells). Scale bar, 20 µm. P-values were calculated using unpaired two-tailed Student's t-test.

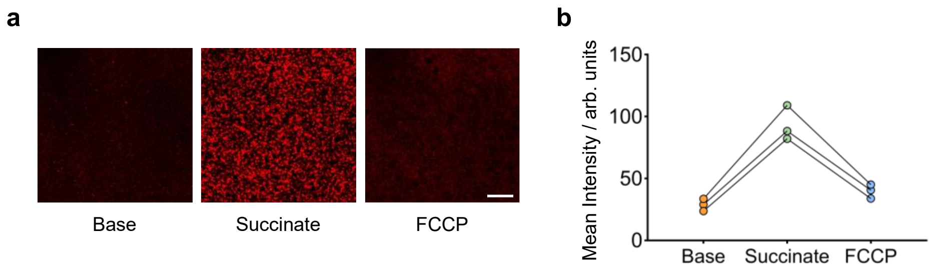


**Supplementary Figure 6**. Confocal imaging of purified mitochondria stained with TMRM and treated with succinate and FCCP (**a**) and corresponding plot (**b**). Scale bar, 20 μm.

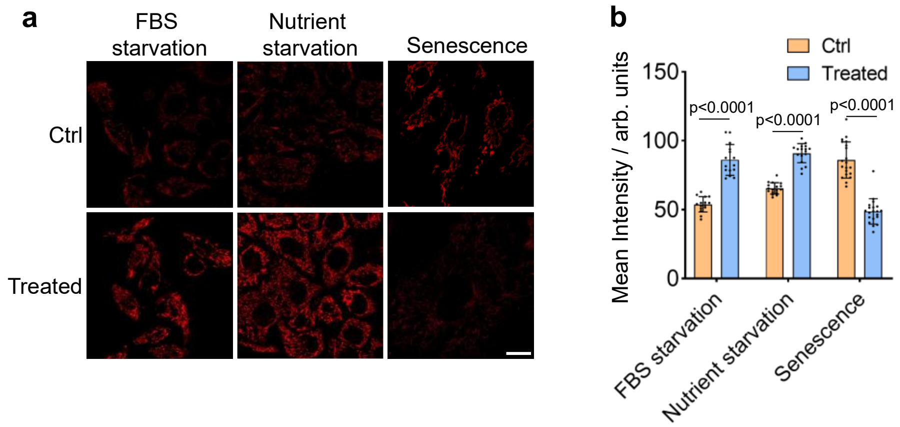


**Supplementary Figure 7**. Mitochondrial membrane potential in starvation and senescence cell. **(a)** TMRM-Intensity images of HeLa cells after FBS starvation and nutrient starvation (cultured in DMEM lack of glucose, sodium-pyruvate and glutamine), and HUVECs at senescence conditions. Scale bar, 20 µm. **(b)** Plots showing the average mitochondrial fluorescence intensity under FBS starvation (n = 15 cells), nutrient starvation (n = 17 cells), and senescence HUVECs (n = 20 cells). P-values were calculated using unpaired two-tailed Student's t-test.

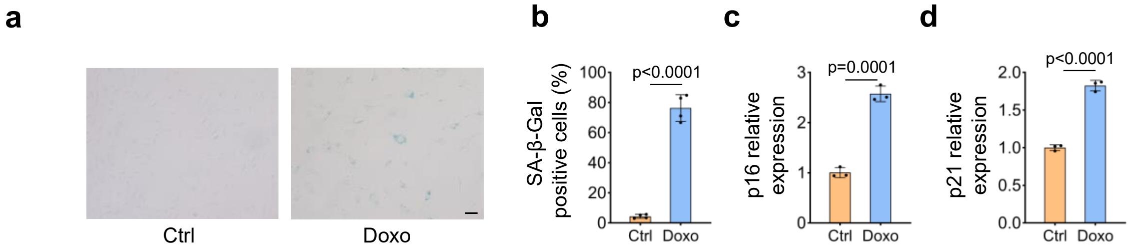


**Supplementary Figure 8**. Doxorubicin induces HUVEC senescence. **(a)** SABG staining of HUVEC, untreated or doxorubicin treatment. **(b)** The percentage of SA-β-Gal positive area (blue ones in **(a)**) compared to the total area was evaluated in three independent experiments, and the results are represented as the mean ± SD **(c-d)** RT-qPCR analysis to measure the expression levels of p16 **(c)** and p21 **(d)** mRNAs. Scale bar, 50 μm. P-values were calculated using unpaired Student's t-test.

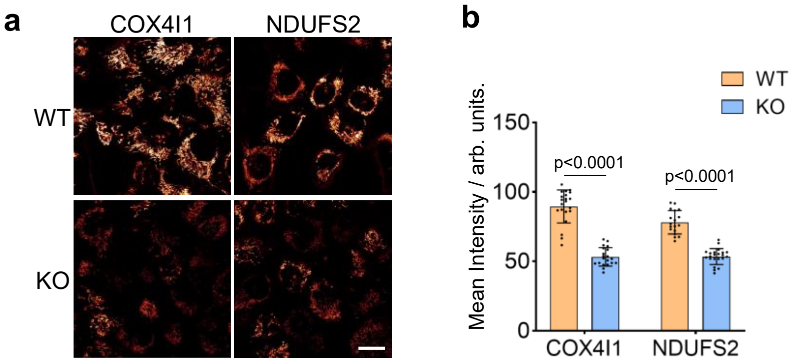


**Supplementary Figure 9**. Mitochondrial membrane potential in COX4I1 and NDUFS2 knockout 143B cells. **(a)** TMRM-intensity images of wild type, COX4I1 and NDUFS2 knockout 143B cells. Scale bar, 20 µm. **(b)** Plots showing the average mitochondrial fluorescence lifetime in wild type (n = 20 cells) and knockout cells (n = 22 cells). P-values were calculated using unpaired Student's t-test.

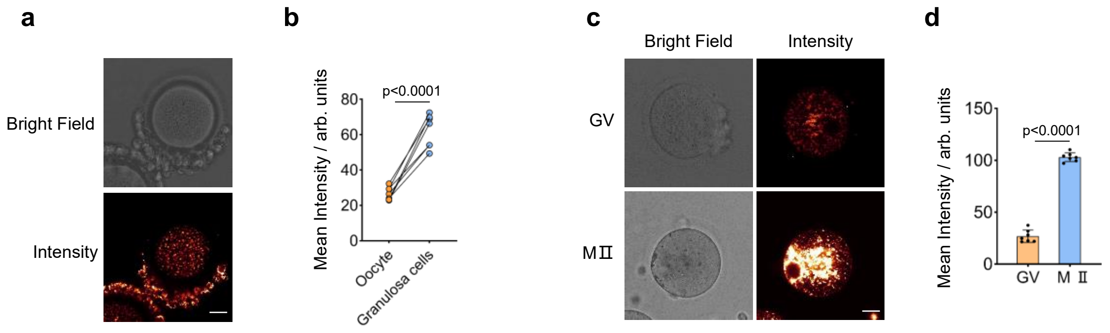


**Supplementary Figure 10**. Mitochondrial membrane potential in oocytes. **(a)** Bright field and confocal images of TMRM-stained mitochondria in GV stage oocyte and granulosa cells. **(b)** Plots showing the average mitochondrial fluorescence lifetime in oocyte and granulosa cells (n = 7 cells). **(c)** Bright field and confocal images of TMRM-stained mitochondria in GV and MⅡ oocyte. **(d)** Plots showing the average mitochondrial fluorescence lifetime in GV and MⅡ oocyte (n = 7 cells). Scale bar, 20 μm.

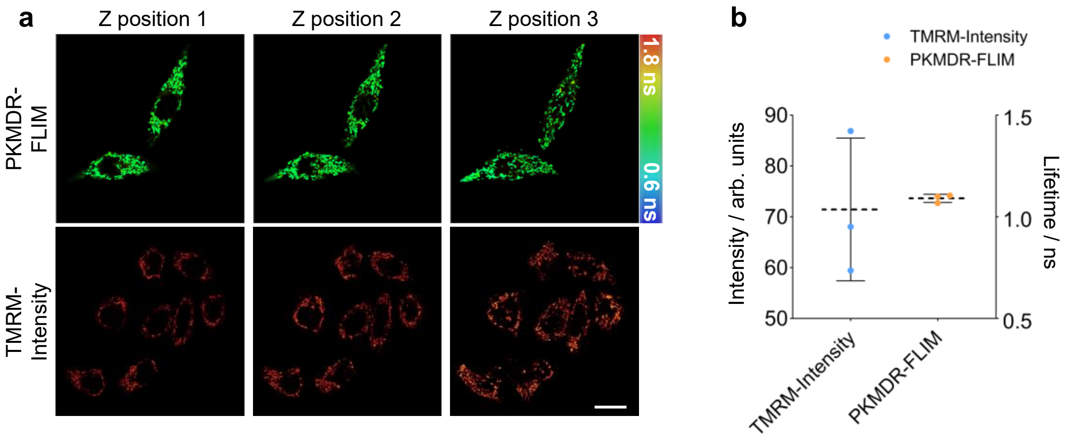


**Supplementary Figure 11.** Measurement of mitochondrial membrane potential using TMRM-intensity and PKMDR-FLIM. **(a)** FLIM (upper) and confocal (lower) image of HeLa cells stained with PKMDR and TMRM. Scale bar, 20 μM. **(b)** Plots showing the mitochondrial fluorescence intensity and lifetime in HeLa cells at different Z position.

# Supplementary Tables

Supplementary Table 1. Comparison of selected mitochondrial dyes.

| Name | Abs/Em (nm) | Quantum Yield | Extinction Coefficient ($M^{-1}\cdot cm^{-1}$) | Brightness | Singlet oxygen quantum yields Units: X $10^{-4}$ (Mean ± SD) | Remark | Ref |
|---|---|---|---|---|---|---|---|
| **PKMDR** | 644/670 (MeOH) | 0.16 (MeOH) | $2.6 \times 10^5$ (MeOH) | $4.16 \times 10^4$ (MeOH) | 2.25±0.13 | Lowest phototoxicity. **FLIM available for MMP detection (this work).** | 1 |
| Rhodamine123 | 508/528 | 0.90 (EtOH) | $8.52 \times 10^4$ (EtOH) | $7.67 \times 10^4$ (EtOH) | 290 ± 7 | Intensity readout for distribution according to $\Delta\psi$ and $\Delta V$. Serious phototoxicity. | 2,3 |
| TMRM | 548/572 (water) | 0.41 (water) | $7.8 \times 10^4$ (water) | $3.2\times 10^4$ (water) | 218 ± 5 | Intensity readout for distribution according to $\Delta\psi$ and $\Delta V$. Relatively high phototoxicity. | 3-5 |
| JC-1 | 510/527 (mono) 585/590 (J agg) | - | $1.287 \times 10^5$ (DMSO, 510 nm) | - | 6.99±0.92 | JC-1 forms "J-aggregate" while accumulating at IMM and undergoes spectra shift from red to green. | 6 |
| Mitorotor-1 | 552/603 (MeOH) | 0.018±0.0002 (MeOH) | $6.9 \times 10^4$ (MeOH) | $0.12 \times 10^4$ (MeOH) | 7.21±1.40 | FLIM result for IMM local viscosity readout which influences the free volume of rotor molecules. Obliquely indicate metabolic state of mitochondria. | 7 8 |
| Mito Flipper-TR | 480/600* (EA) | 0.30 (EA) | $1.6 \times 10^4$ (EA) | $0.48 \times 10^4$ (EA) | - | Monitor IMM tension and fission. | 9 10 |
| MitoPB Red | 491/638 ($CH_2Cl_2$) | 0.52 ($CH_2Cl_2$) | $1.76 \times 10^4$ ($CH_2Cl_2$) | $0.92 \times 10^4$ ($CH_2Cl_2$) | - | STED available. FLIM available and shows good sensitivity to membrane order. | 11 |

Supplementary Table 2. Fluorescence microscopy data acquisition parameters.

| Image | Microscpe | Excitation (nm) | Objective | Scan speed (Hz) | Zoom | Pixel Size (nm) | Size (pixels) | Comment |
|---|---|---|---|---|---|---|---|---|
| Fig.1 e-f | SP8 | 633 | 100×1.40 oil | 100 | 1 | 114 | 1024×1024 | bi-exponential fits, 500 photons |
| Fig.2 c | SP8 | 633 | 100×1.40 oil | 200 | 1 | 227 | 512×512 | tri-exponential fits, 10 frame accumulation |
| Fig. 2 e | SP8 | 633 | 100×1.40 oil | 400 | 1 | 227 | 512×512 | tri-exponential fits, 500 photons, time-lapse inverval: 15s |
| Fig.3 a | SP8 | 633 | 100×1.40 oil | 400 | 1 | 227 | 512×512 | tri-exponential fits, 10 frame accumulation |
| Fig.3 d | SP8 | 633 | 100×1.40 oil | 400 | 1 | 227 | 512×512 | tri-exponential fits, 10 frame accumulation |
| Fig.3 f | STELLARIS 8 FALCON | 638 | 40x1.30 oil | 100 | 2 | 142 | 1024×1024 | tri-exponential fits, 5 frame accumulation |
| Fig.3 h | STELLARIS 8 FALCON | 638 | 40x1.30 oil | 100 | 2 | 142 | 1024×1024 | tri-exponential fits, 5 frame accumulation |
| Fig.3 j | SP8 | 633 | 100×1.40 oil | 400 | 1 | 227 | 512×512 | tri -exponential fits, 10 frame accumulation |
| Fig.4 b | STELLARIS 8 FALCON | 638 | 40x1.30 oil | 100 | 2 | 142 | 1024×1024 | tri-exponential fits, 5 frame accumulation |
| Fig.5 a-b | STELLARIS 8 FALCON | 638 | 40x1.30 oil | 100 | 2 | 142 | 1024×1024 | tri-exponential fits, 5 frame accumulation |
| Fig.5 c | SP8 | 633 | 100×1.40 oil | 400 | 1 | 227 | 512×512 | bi-exponential fits, 10 frame accumulation |
| Fig.5 d-e | SP8 | 633 | 100×1.40 oil | 100 | 1 | 114 | 1024×1024 | bi-exponential fits, 500 photons |
| Fig.6 a | STELLARIS 8 FALCON | 638 | 63×1.40 oil | 400 | 1 | 361 | 512×512 | tri-exponential fits, 5 frame accumulation, time-lapse inverval: 15s |
| Fig.6 c | SP8 | 633 | 100×1.40 oil | 100 | 1 | 114 | 1024×1024 | tri-exponential fits, 500 photons, time-lapse inverval: 11s |

# Supplementary Information Text

## Synthesis and Characterization of Mitorotor-1

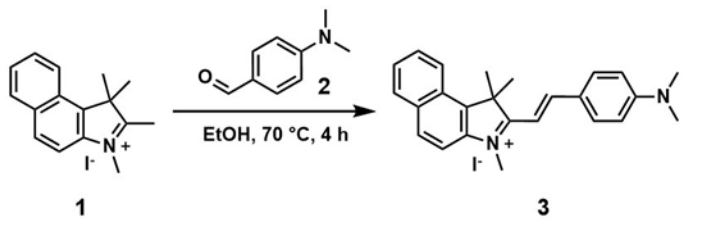


Compound 3 was prepared by a slight modification of the previous reported procedure[7]. 1,1,2,3-Tetramethyl-1*H*-benz[*e*]indolium iodide (Compound 1; 20 mg, 0.057 mmol) and 4-(dimethylamino) benzaldehyde (Compound 2; 25 mg, 0.085 mmol, 1.5 eq) were added in EtOH (5 mL). The resulted mixture was allowed to stirred at 70 °C for 4 h in a sealed tube. After cooling to room temperature, the solvent was removed under reduced pressure and the residue was purified by HPLC (eluent, a 30 min linear gradient, from 30% to 90% solvent B; flow rate, 10 mL/min; detection wavelength, 550 nm; eluent A (dd$H_2O$) and eluent B ($CH_3CN$)) to obtain compound 3 (12 mg, 0.025 mmol, 44% yield) as a brick red solid.

$^1$H NMR (400 MHz, Methanol-$d_4$) δ 8.39 (d, $J$ = 15.7 Hz, 1H), 8.34 (d, $J$ = 8.5 Hz, 1H), 8.15 (d, $J$ = 8.9 Hz, 1H), 8.10 (d, $J$ = 8.3 Hz, 1H), 7.95 (d, $J$ = 9.0 Hz, 2H), 7.83 (d, $J$ = 8.9 Hz, 1H), 7.74 (ddd, $J$ = 8.4, 6.9, 1.3 Hz, 1H), 7.62 (ddd, $J$ = 8.0, 6.9, 1.0 Hz, 1H), 7.26 (d, $J$ = 15.7 Hz, 1H), 6.91 (d, $J$ = 9.2 Hz, 2H), 4.09 (s, 3H), 3.21 (s, 6H), 2.06 (s, 6H).

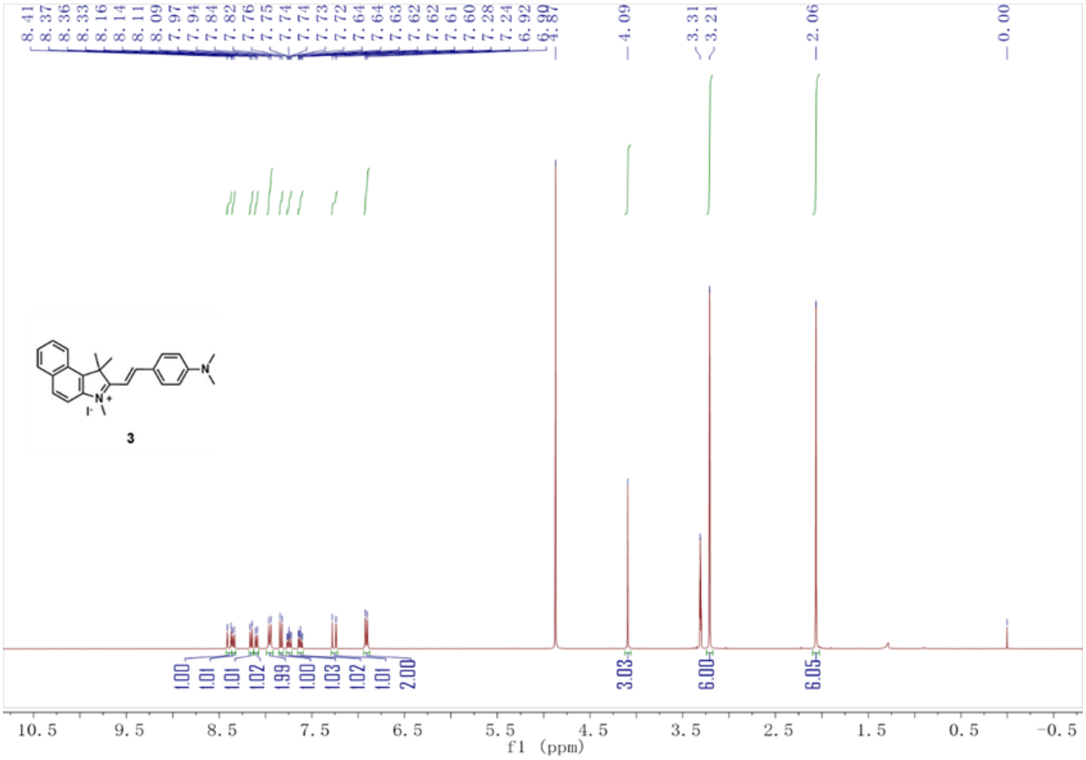


$^{13}$C NMR (101 MHz, MeOD) δ 182.68, 156.53, 155.38, 140.86, 137.81, 134.93, 134.65, 132.20, 131.25, 129.27, 128.84, 127.58, 123.81, 113.55, 112.94, 105.21, 54.28, 40.35, 33.67, 26.94. MS (ESI) calculated for $C_{25}H_{27}N_2$ ($M^+$) 355.2, observed 355.9.

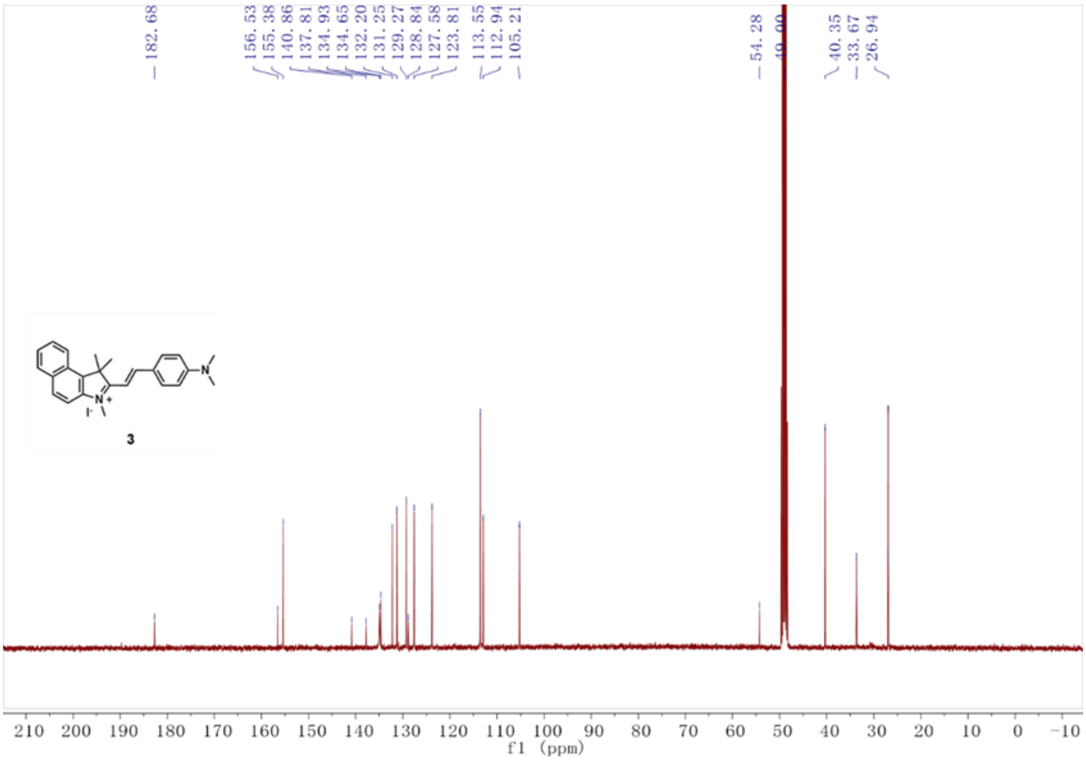

**Data Availability**

All data associated with this study are presented in the main text or Supplementary Information. The raw numbers for charts and graphs are available in the Source Data. Source data are provided with this paper.

# References


1 Yang, Z. *et al.* Cyclooctatetraene-conjugated cyanine mitochondrial probes minimize phototoxicity in fluorescence and nanoscopic imaging. *Chem Sci* **11**, 8506-8516, (2020).

2 Kubin, R. F. & Fletcher, A. N. Fluorescence quantum yields of some rhodamine dyes. *Journal of Luminescence* **27**, 455-462, (1982).

3 Liu, T. *et al.* Gentle Rhodamines for Live-Cell Fluorescence Microscopy. *ACS Central Science* **10**, 1933-1944, (2024).

4 Desai, S. *et al.* Performance of TMRM and Mitotrackers in mitochondrial morphofunctional analysis of primary human skin fibroblasts. *Biochimica et Biophysica Acta (BBA) - Bioenergetics* **1865**, 149027, (2024).

5 Grimm, J. B. *et al.* A general method to improve fluorophores for live-cell and single-molecule microscopy. *Nature Methods* **12**, 244-250, (2015).

6 Smiley, S. T. *et al.* Intracellular heterogeneity in mitochondrial membrane potentials revealed by a J-aggregate-forming lipophilic cation JC-1. **88**, 3671-3675, (1991).

7 Singh, G. *et al.* A molecular rotor FLIM probe reveals dynamic coupling between mitochondrial inner membrane fluidity and cellular respiration. *Proc Natl Acad Sci U S A* **120**, e2213241120, (2023).

8 Raja, S. O., Sivaraman, G., Mukherjee, A., Duraisamy, C. & Gulyani, A. Facile Synthesis of Highly Sensitive, Red-Emitting, Fluorogenic Dye for Microviscosity and Mitochondrial Imaging in Embryonic Stem Cells. *ChemistrySelect* **2**, 4609-4616, (2017).

9 Goujon, A. *et al.* Mechanosensitive Fluorescent Probes to Image Membrane Tension in Mitochondria, Endoplasmic Reticulum, and Lysosomes. *Journal of the American Chemical Society* **141**, 3380-3384, (2019).

10 Mahecic, D. *et al.* Mitochondrial membrane tension governs fission. *Cell reports* **35**, 108947, (2021).

11 Wang, J., Taki, M., Ohba, Y., Arita, M. & Yamaguchi, S. Fluorescence Lifetime Imaging of Lipid Heterogeneity in the Inner Mitochondrial Membrane with a Super - photostable Environment - Sensitive Probe. *Angewandte Chemie International Edition* **63**, (2024).
