# Supplementary material for: Imaging mitochondrial membrane potential via concentration-dependent fluorescence lifetime changes
Source: Nat Commun. 2025 Dec 12;16:11088. doi: 10.1038/s41467-025-66042-x (PMC12700901; doi:10.1038/s41467-025-66042-x)
Supplement: Supplementary file 2 — Reporting Summary [file 41467_2025_66042_MOESM2_ESM.pdf]

nature portfolio

Corresponding author(s): Zhixing Chen
Last updated by author(s): Oct 3, 2025

Reporting Summary

Nature Portfolio wishes to improve the reproducibility of the work that we publish. This form provides structure for consistency and transparency in reporting. For further information on Nature Portfolio policies, see our Editorial Policies and the Editorial Policy Checklist.

Statistics

For all statistical analyses, confirm that the following items are present in the figure legend, table legend, main text, or Methods section.

n/a	Confirmed	
☐	☒	The exact sample size (*n*) for each experimental group/condition, given as a discrete number and unit of measurement
☐	☒	A statement on whether measurements were taken from distinct samples or whether the same sample was measured repeatedly
☐	☒	The statistical test(s) used AND whether they are one- or two-sided<br>*Only common tests should be described solely by name; describe more complex techniques in the Methods section.*
☒	☐	A description of all covariates tested
☒	☐	A description of any assumptions or corrections, such as tests of normality and adjustment for multiple comparisons
☐	☒	A full description of the statistical parameters including central tendency (e.g. means) or other basic estimates (e.g. regression coefficient) AND variation (e.g. standard deviation) or associated estimates of uncertainty (e.g. confidence intervals)
☐	☒	For null hypothesis testing, the test statistic (e.g. *F*, *t*, *r*) with confidence intervals, effect sizes, degrees of freedom and *P* value noted<br>*Give P values as exact values whenever suitable.*
☒	☐	For Bayesian analysis, information on the choice of priors and Markov chain Monte Carlo settings
☒	☐	For hierarchical and complex designs, identification of the appropriate level for tests and full reporting of outcomes
☒	☐	Estimates of effect sizes (e.g. Cohen's *d*, Pearson's *r*), indicating how they were calculated

Our web collection on statistics for biologists contains articles on many of the points above.

Software and code

Policy information about availability of computer code

Data collection	Edinburgh Fluorade, Leica LAS X
Data analysis	GraphPad Prism 9.5.1, Fiji 2.0.0, Leica LAS X, Fluoracle

For manuscripts utilizing custom algorithms or software that are central to the research but not yet described in published literature, software must be made available to editors and reviewers. We strongly encourage code deposition in a community repository (e.g. GitHub). See the Nature Portfolio guidelines for submitting code & software for further information.

Data

Policy information about availability of data

All manuscripts must include a data availability statement. This statement should provide the following information, where applicable:
- Accession codes, unique identifiers, or web links for publicly available datasets
- A description of any restrictions on data availability
- For clinical datasets or third party data, please ensure that the statement adheres to our policy

All data associated with this study are presented in the main text or Supplementary Information. The raw numbers for charts and graphs are available in the Source Data. Source data are provided with this paper.

Research involving human participants, their data, or biological material

Policy information about studies with human participants or human data. See also policy information about sex, gender (identity/presentation), and sexual orientation and race, ethnicity and racism.

Reporting on sex and gender	N/A
Reporting on race, ethnicity, or other socially relevant groupings	N/A
Population characteristics	N/A
Recruitment	N/A
Ethics oversight	N/A

Note that full information on the approval of the study protocol must also be provided in the manuscript.

Field-specific reporting

Please select the one below that is the best fit for your research. If you are not sure, read the appropriate sections before making your selection.

☒ Life sciences ☐ Behavioural & social sciences ☐ Ecological, evolutionary & environmental sciences

For a reference copy of the document with all sections, see nature.com/documents/nr-reporting-summary-flat.pdf

Life sciences study design

All studies must disclose on these points even when the disclosure is negative.

Sample size	No statistical method was used for sample size determination. More than two independent replicates of each experiments were performed. This sample size was resulted in low data variability. All quantitative imaging experiments were performed on replicate samples of fields of view as reported in Figure legends and supplementary materials.
Data exclusions	No data exclusion was performed.
Replication	The numbers of replicated experiments are indicated in the main text and figure legends.
Randomization	Labeled cells were chosen in random in imaging experiments.
Blinding	No manual assessment of images was performed.

Reporting for specific materials, systems and methods

We require information from authors about some types of materials, experimental systems and methods used in many studies. Here, indicate whether each material, system or method listed is relevant to your study. If you are not sure if a list item applies to your research, read the appropriate section before selecting a response.

Materials & experimental systems

n/a	Involved in the study	
☒	☐	Antibodies
☐	☒	Eukaryotic cell lines
☒	☐	Palaeontology and archaeology
☐	☒	Animals and other organisms
☒	☐	Clinical data
☒	☐	Dual use research of concern
☒	☐	Plants

Methods

n/a	Involved in the study	
☒	☐	ChIP-seq
☒	☐	Flow cytometry
☒	☐	MRI-based neuroimaging

Eukaryotic cell lines

Policy information about cell lines and Sex and Gender in Research

Cell line source(s)	HeLa,Procell, CL-0101<br>COS-7, Procell, CL-0069<br>L929, Procell, CL-0173<br>143B, COX4I1-KO 143B, NDUFS2-KO 143B, gift from Prof.Hui Jiang's Lab, NIBS

	HUVEC, gift from Prof.Yangming Wang's Lab, Peking University<br>MC38, gift from Prof.Ruiping Xiao's Lab, Peking University<br>U-2 OS Tomm20-GFP, gift from Guangzhou CSR Biotech Co. Ltd<br>MC38 H2B-GFP, made by our Lab; HeLa cells were integrated with an expressing plasmid via the lenti virus.
Authentication	The cell lines were used without further authentication. Genetically modified cell lines clearly exhibited the expected labeling pattern.
Mycoplasma contamination	All cell lines tested negative for mycoplasma contamination.
Commonly misidentified lines<br>(See ICLAC register)	No commonly misidentified cell lines were used in this study.

Animals and other research organisms

Policy information about studies involving animals; ARRIVE guidelines recommended for reporting animal research, and Sex and Gender in Research

Laboratory animals	3-week-old and 8-week-old C57BL/6N mice were used in this study. All animals were purchased from Beijing Vital River Laboratory Animal Technology. The facility maintained a 12-hour light-dark cycle, with a room temperature of 20-24°C and relative humidity of 45-65%.
Wild animals	No wild animals were used in this study.
Reporting on sex	Both male and female mice were used, sex was not considered in the experimental design.
Field-collected samples	No field-collected samples were used.
Ethics oversight	All procedures were approved by the Peking University Animal Use and Care<br>Committee and complied with the standards of the Association for Assessment and<br>Accreditation of Laboratory Animal Care

Note that full information on the approval of the study protocol must also be provided in the manuscript.

Plants

Seed stocks	N/A
Novel plant genotypes	N/A
Authentication	N/A
