# Supplementary material for: Imaging mitochondrial membrane potential via concentration-dependent fluorescence lifetime changes
Source: Nat Commun. 2025 Dec 12;16:11088. doi: 10.1038/s41467-025-66042-x (PMC12700901; doi:10.1038/s41467-025-66042-x)
Supplement: Supplementary file 3 — Description of Additional Supplementary Files [file 41467_2025_66042_MOESM3_ESM.pdf]

Title: Supplementary Movie 1

Description: Long-term time-lapse FLIM imaging of purified mitochondria treated with succinate and FCCP. Scale bar = 20 μm

Title: Supplementary Movie 2

Description: Long-term time-lapse FLIM imaging of L929 cells. Scale bar = 20 μm.

Title: Supplementary Movie 3

Description: Long-term time-lapse FLIM imaging of mitochondria in neurons. Scale bar = 20 μm.
