# Supplementary figures and images for: Imaging mitochondrial membrane potential via concentration-dependent fluorescence lifetime changes (part 2 of 2)
Source: Nat Commun. 2025 Dec 12;16:11088. doi: 10.1038/s41467-025-66042-x (PMC12700901; doi:10.1038/s41467-025-66042-x)

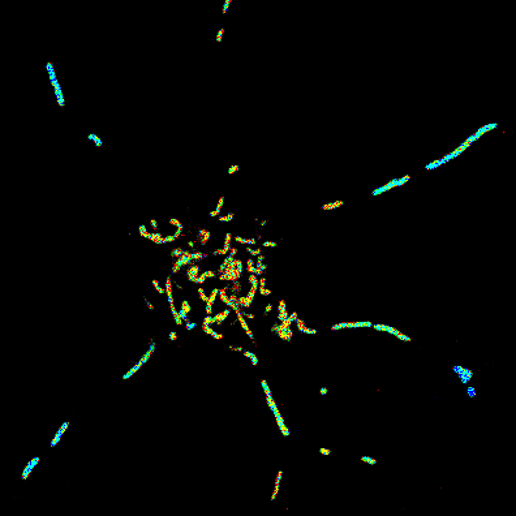

Supplement: Supplementary file 8 — Source Data [file 41467_2025_66042_MOESM8_ESM.zip › Source data/Figure 6/6c-3.tif]

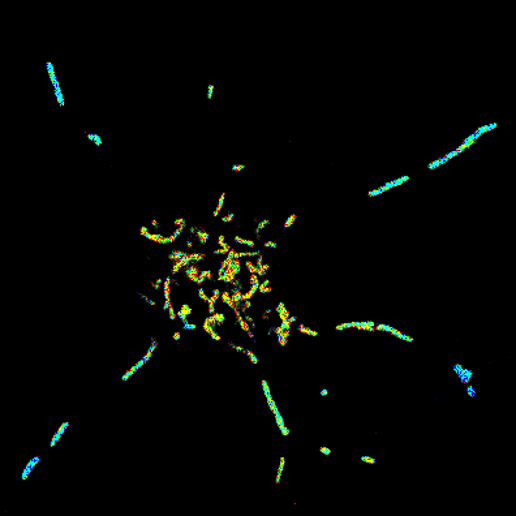

Supplement: Supplementary file 8 — Source Data [file 41467_2025_66042_MOESM8_ESM.zip › Source data/Figure 6/6c-4.tif]

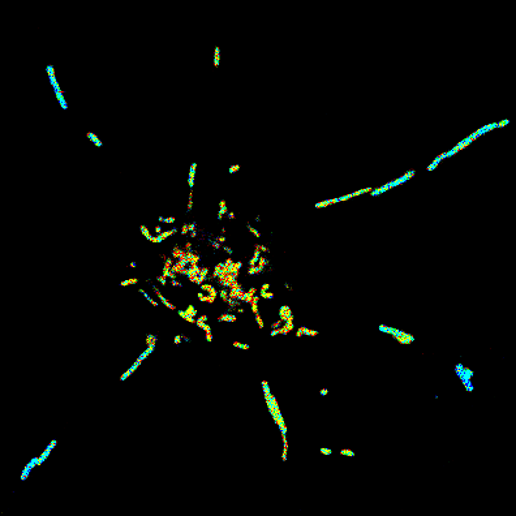

Supplement: Supplementary file 8 — Source Data [file 41467_2025_66042_MOESM8_ESM.zip › Source data/Figure 6/6c-5.tif]

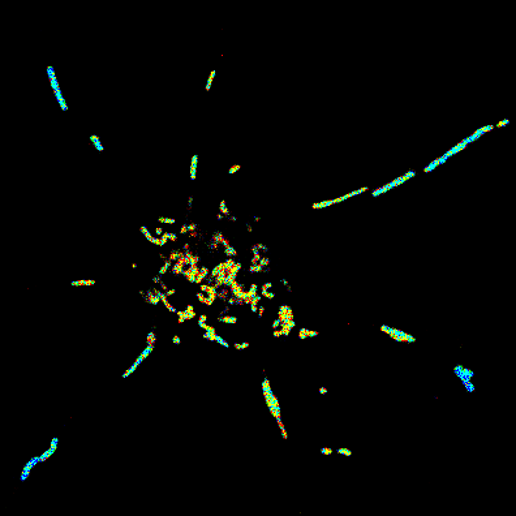

Supplement: Supplementary file 8 — Source Data [file 41467_2025_66042_MOESM8_ESM.zip › Source data/Figure 6/6c-6.tif]

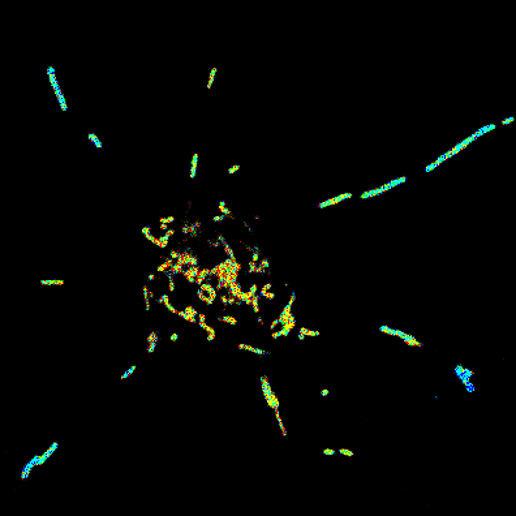

Supplement: Supplementary file 8 — Source Data [file 41467_2025_66042_MOESM8_ESM.zip › Source data/Figure 6/6c-7.tif]
