# Supplementary figures and images for: Imaging mitochondrial membrane potential via concentration-dependent fluorescence lifetime changes (part 1 of 2)
Source: Nat Commun. 2025 Dec 12;16:11088. doi: 10.1038/s41467-025-66042-x (PMC12700901; doi:10.1038/s41467-025-66042-x)

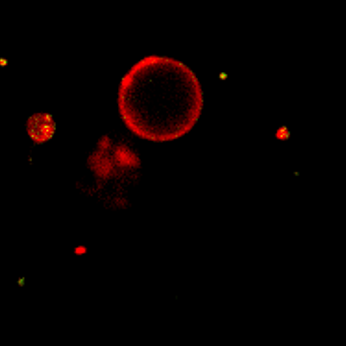

Supplement: Supplementary file 8 — Source Data [file 41467_2025_66042_MOESM8_ESM.zip › Source data/Figure 1/Mitorotor-1 100nM.tif]

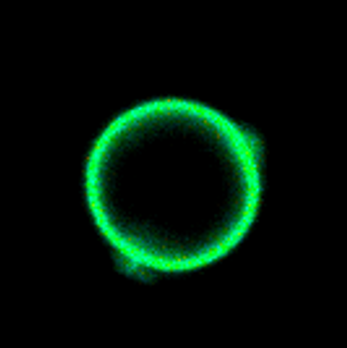

Supplement: Supplementary file 8 — Source Data [file 41467_2025_66042_MOESM8_ESM.zip › Source data/Figure 1/Mitorotor-1 10uM.tif]

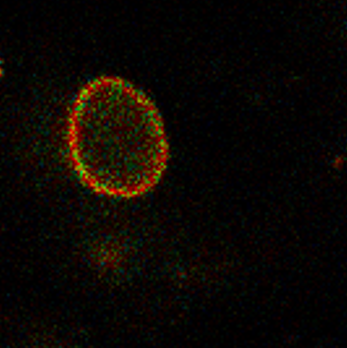

Supplement: Supplementary file 8 — Source Data [file 41467_2025_66042_MOESM8_ESM.zip › Source data/Figure 1/Mitorotor-1 1uM.tif]

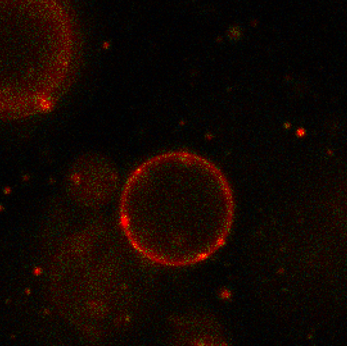

Supplement: Supplementary file 8 — Source Data [file 41467_2025_66042_MOESM8_ESM.zip › Source data/Figure 1/Mitorotor-1 200nM.tif]

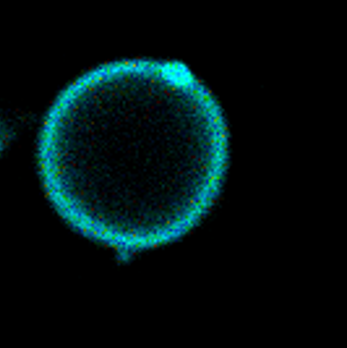

Supplement: Supplementary file 8 — Source Data [file 41467_2025_66042_MOESM8_ESM.zip › Source data/Figure 1/Mitorotor-1 20uM.tif]

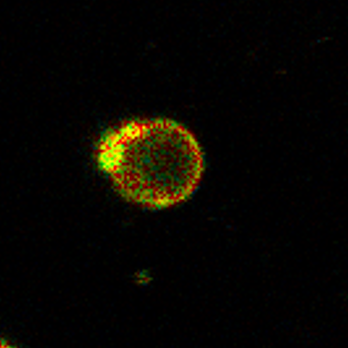

Supplement: Supplementary file 8 — Source Data [file 41467_2025_66042_MOESM8_ESM.zip › Source data/Figure 1/Mitorotor-1 2uM.tif]

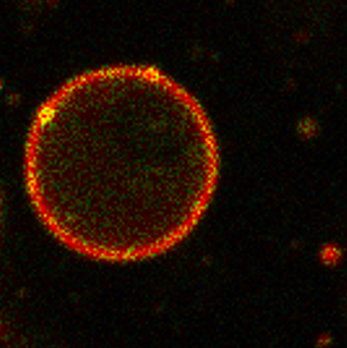

Supplement: Supplementary file 8 — Source Data [file 41467_2025_66042_MOESM8_ESM.zip › Source data/Figure 1/Mitorotor-1 500nM.tif]

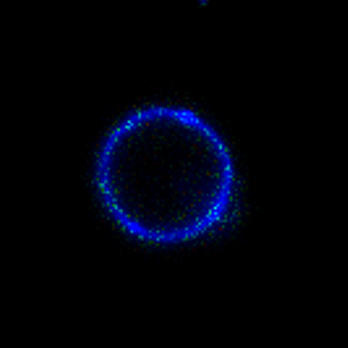

Supplement: Supplementary file 8 — Source Data [file 41467_2025_66042_MOESM8_ESM.zip › Source data/Figure 1/Mitorotor-1 50uM.tif]

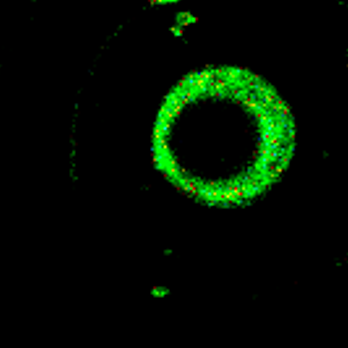

Supplement: Supplementary file 8 — Source Data [file 41467_2025_66042_MOESM8_ESM.zip › Source data/Figure 1/Mitorotor-1 5uM.tif]

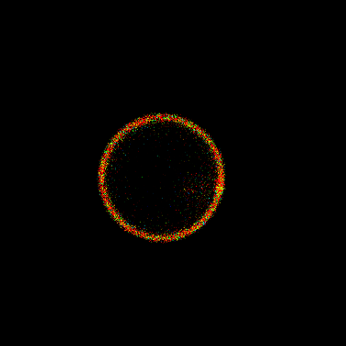

Supplement: Supplementary file 8 — Source Data [file 41467_2025_66042_MOESM8_ESM.zip › Source data/Figure 1/PKMDR 100nM.tif]

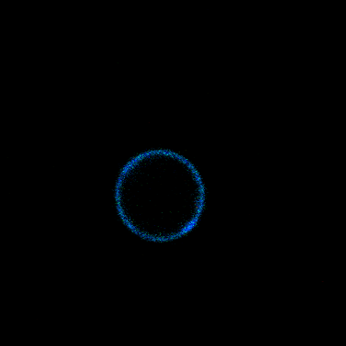

Supplement: Supplementary file 8 — Source Data [file 41467_2025_66042_MOESM8_ESM.zip › Source data/Figure 1/PKMDR 10uM.tif]

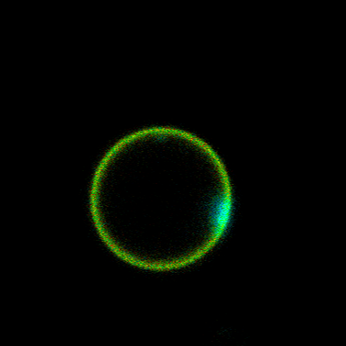

Supplement: Supplementary file 8 — Source Data [file 41467_2025_66042_MOESM8_ESM.zip › Source data/Figure 1/PKMDR 1uM.tif]

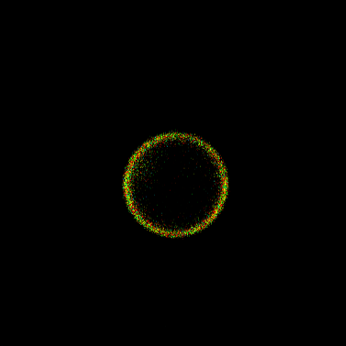

Supplement: Supplementary file 8 — Source Data [file 41467_2025_66042_MOESM8_ESM.zip › Source data/Figure 1/PKMDR 200nM.tif]

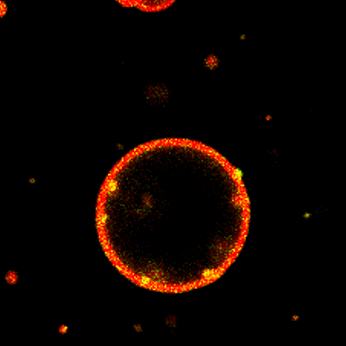

Supplement: Supplementary file 8 — Source Data [file 41467_2025_66042_MOESM8_ESM.zip › Source data/Figure 1/PKMDR 20nM.tif]

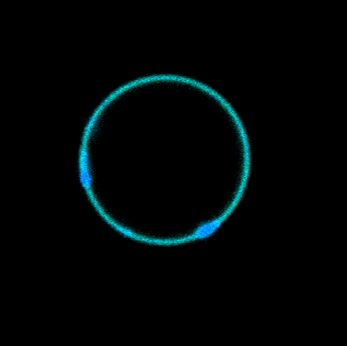

Supplement: Supplementary file 8 — Source Data [file 41467_2025_66042_MOESM8_ESM.zip › Source data/Figure 1/PKMDR 2uM.tif]

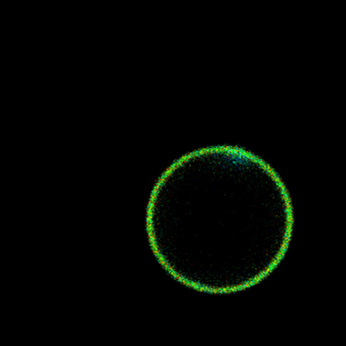

Supplement: Supplementary file 8 — Source Data [file 41467_2025_66042_MOESM8_ESM.zip › Source data/Figure 1/PKMDR 500nM.tif]

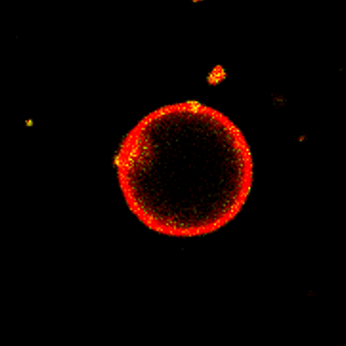

Supplement: Supplementary file 8 — Source Data [file 41467_2025_66042_MOESM8_ESM.zip › Source data/Figure 1/PKMDR 50nM.tif]

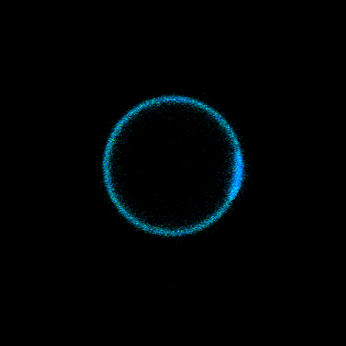

Supplement: Supplementary file 8 — Source Data [file 41467_2025_66042_MOESM8_ESM.zip › Source data/Figure 1/PKMDR 5uM.tif]

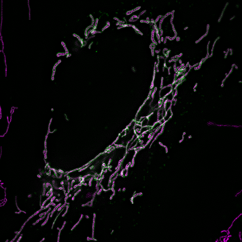

Supplement: Supplementary file 8 — Source Data [file 41467_2025_66042_MOESM8_ESM.zip › Source data/Figure 2/2a.tif]

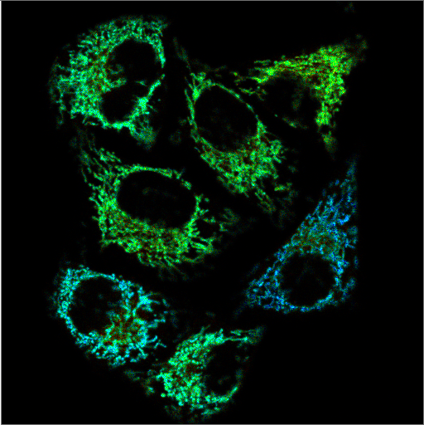

Supplement: Supplementary file 8 — Source Data [file 41467_2025_66042_MOESM8_ESM.zip › Source data/Figure 2/2c-1.tif]

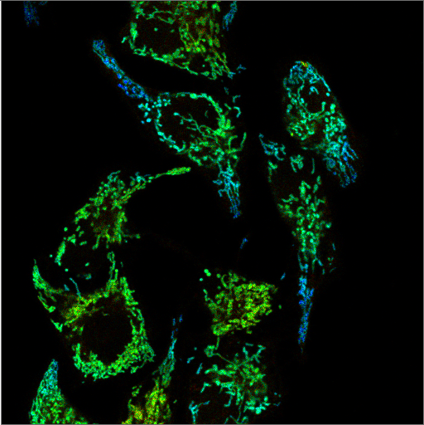

Supplement: Supplementary file 8 — Source Data [file 41467_2025_66042_MOESM8_ESM.zip › Source data/Figure 2/2c-2.tif]

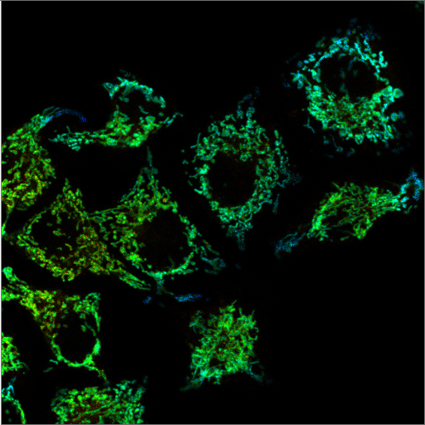

Supplement: Supplementary file 8 — Source Data [file 41467_2025_66042_MOESM8_ESM.zip › Source data/Figure 2/2c-3.tif]

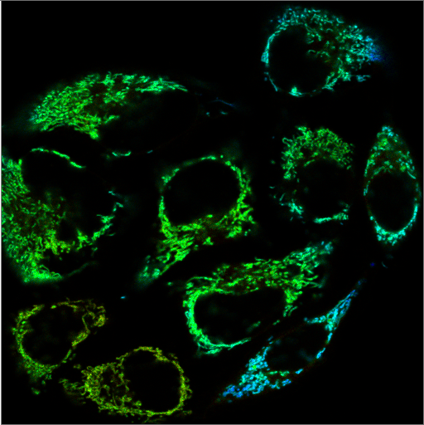

Supplement: Supplementary file 8 — Source Data [file 41467_2025_66042_MOESM8_ESM.zip › Source data/Figure 2/2c-4.tif]

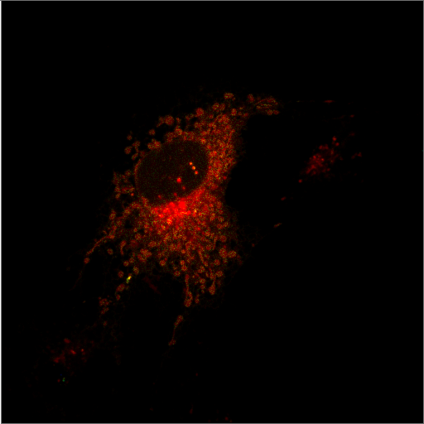

Supplement: Supplementary file 8 — Source Data [file 41467_2025_66042_MOESM8_ESM.zip › Source data/Figure 2/2c-5.tif]

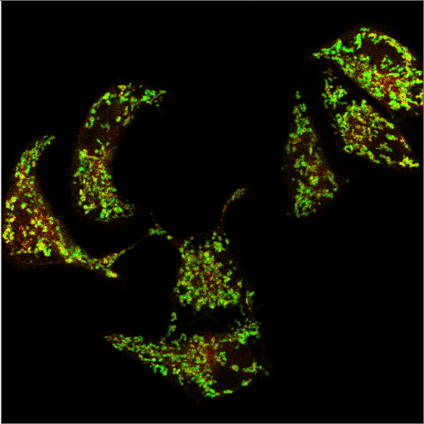

Supplement: Supplementary file 8 — Source Data [file 41467_2025_66042_MOESM8_ESM.zip › Source data/Figure 2/2c-6.tif]

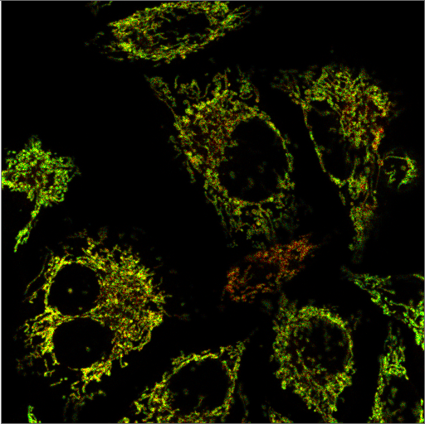

Supplement: Supplementary file 8 — Source Data [file 41467_2025_66042_MOESM8_ESM.zip › Source data/Figure 2/2c-7.tif]

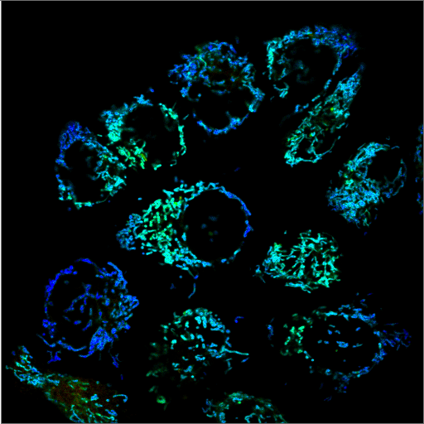

Supplement: Supplementary file 8 — Source Data [file 41467_2025_66042_MOESM8_ESM.zip › Source data/Figure 2/2c-8.tif]

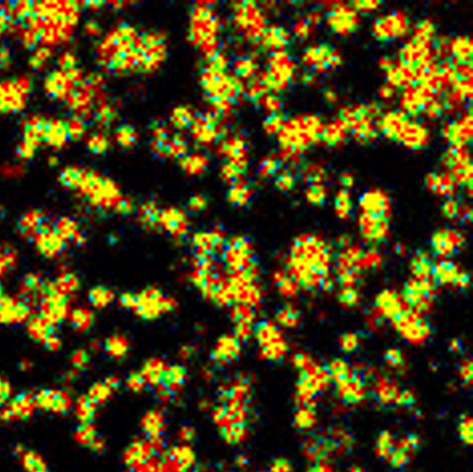

Supplement: Supplementary file 8 — Source Data [file 41467_2025_66042_MOESM8_ESM.zip › Source data/Figure 2/2e-1.tif]

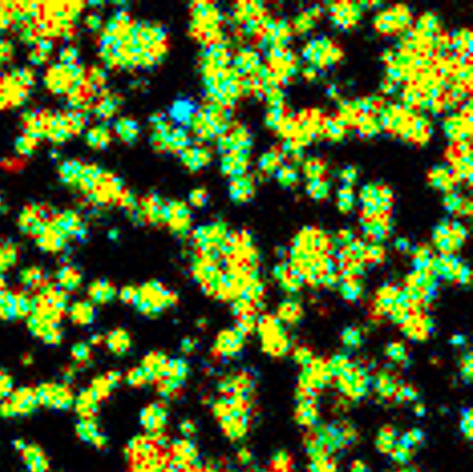

Supplement: Supplementary file 8 — Source Data [file 41467_2025_66042_MOESM8_ESM.zip › Source data/Figure 2/2e-2.tif]

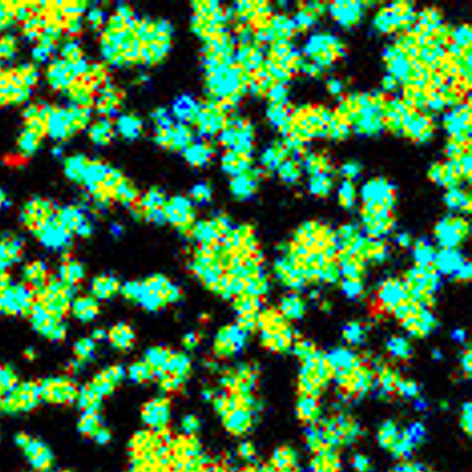

Supplement: Supplementary file 8 — Source Data [file 41467_2025_66042_MOESM8_ESM.zip › Source data/Figure 2/2e-3.tif]

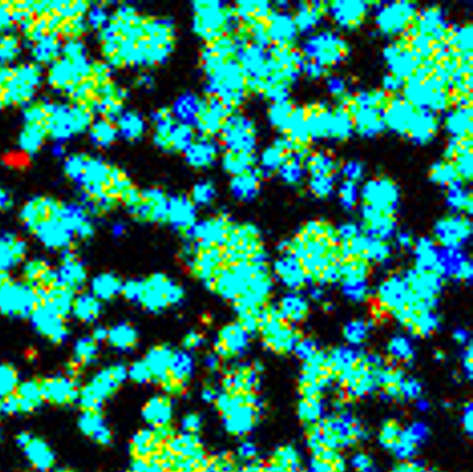

Supplement: Supplementary file 8 — Source Data [file 41467_2025_66042_MOESM8_ESM.zip › Source data/Figure 2/2e-4.tif]

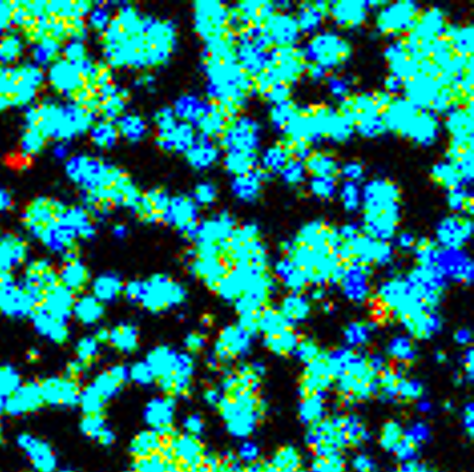

Supplement: Supplementary file 8 — Source Data [file 41467_2025_66042_MOESM8_ESM.zip › Source data/Figure 2/2e-5.tif]

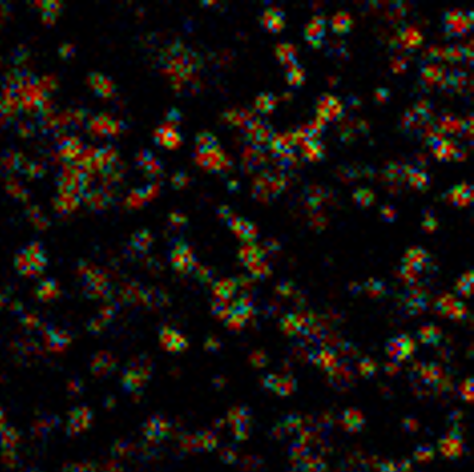

Supplement: Supplementary file 8 — Source Data [file 41467_2025_66042_MOESM8_ESM.zip › Source data/Figure 2/2e-6.tif]

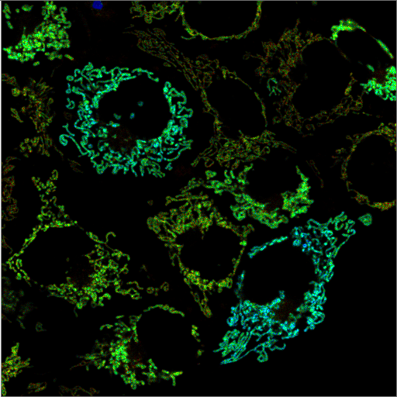

Supplement: Supplementary file 8 — Source Data [file 41467_2025_66042_MOESM8_ESM.zip › Source data/Figure 3/3a-1.tif]

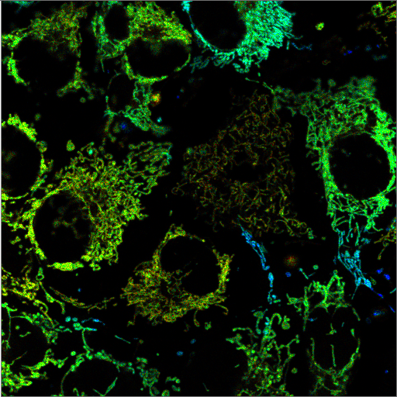

Supplement: Supplementary file 8 — Source Data [file 41467_2025_66042_MOESM8_ESM.zip › Source data/Figure 3/3a-2.tif]

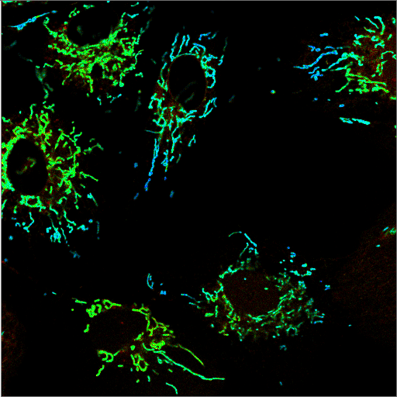

Supplement: Supplementary file 8 — Source Data [file 41467_2025_66042_MOESM8_ESM.zip › Source data/Figure 3/3a-3.tif]

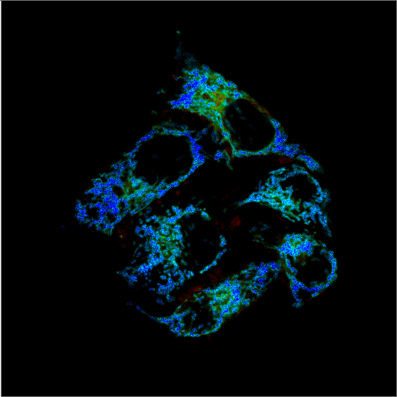

Supplement: Supplementary file 8 — Source Data [file 41467_2025_66042_MOESM8_ESM.zip › Source data/Figure 3/3a-4.tif]

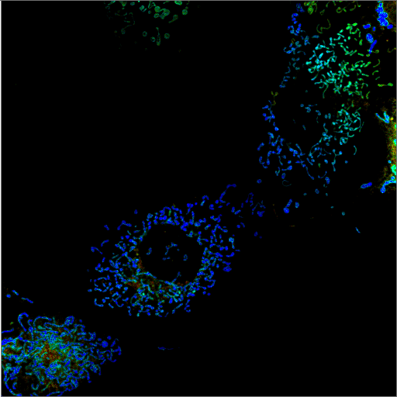

Supplement: Supplementary file 8 — Source Data [file 41467_2025_66042_MOESM8_ESM.zip › Source data/Figure 3/3a-5.tif]

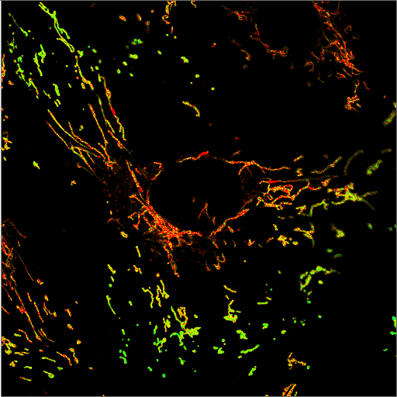

Supplement: Supplementary file 8 — Source Data [file 41467_2025_66042_MOESM8_ESM.zip › Source data/Figure 3/3a-6.tif]

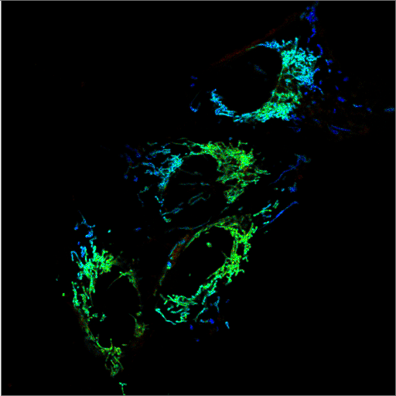

Supplement: Supplementary file 8 — Source Data [file 41467_2025_66042_MOESM8_ESM.zip › Source data/Figure 3/3d-1.tif]

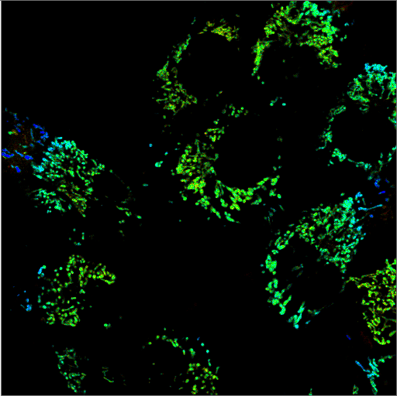

Supplement: Supplementary file 8 — Source Data [file 41467_2025_66042_MOESM8_ESM.zip › Source data/Figure 3/3d-2.tif]

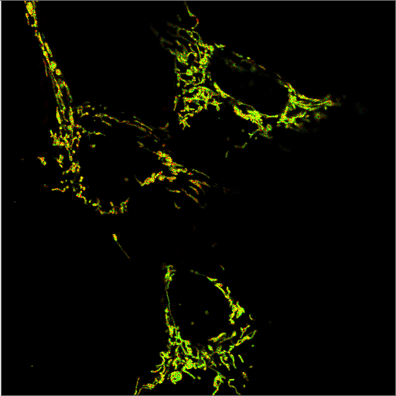

Supplement: Supplementary file 8 — Source Data [file 41467_2025_66042_MOESM8_ESM.zip › Source data/Figure 3/3d-3.tif]

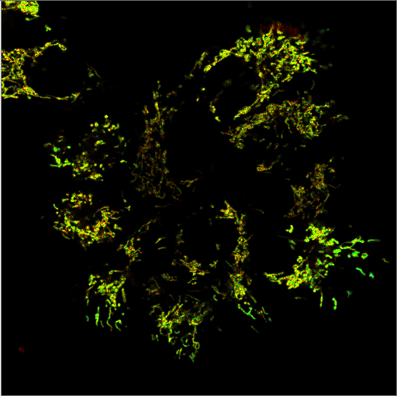

Supplement: Supplementary file 8 — Source Data [file 41467_2025_66042_MOESM8_ESM.zip › Source data/Figure 3/3d-4.tif]

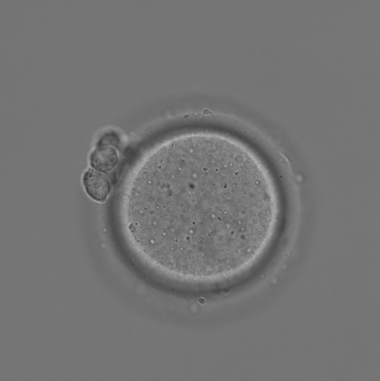

Supplement: Supplementary file 8 — Source Data [file 41467_2025_66042_MOESM8_ESM.zip › Source data/Figure 3/3f-1.tif]

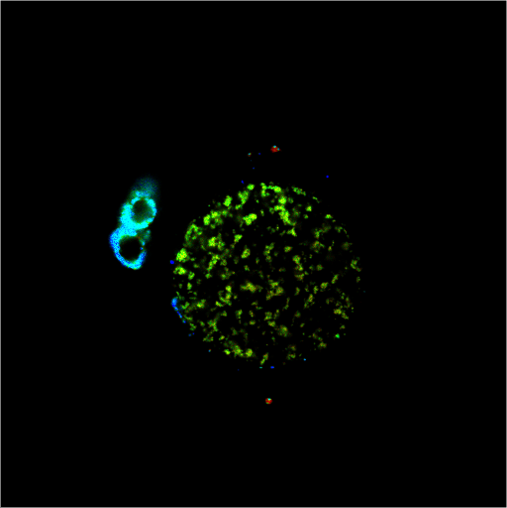

Supplement: Supplementary file 8 — Source Data [file 41467_2025_66042_MOESM8_ESM.zip › Source data/Figure 3/3f-2.tif]

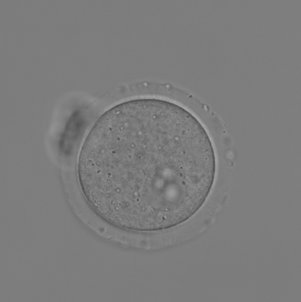

Supplement: Supplementary file 8 — Source Data [file 41467_2025_66042_MOESM8_ESM.zip › Source data/Figure 3/3h-1.tif]

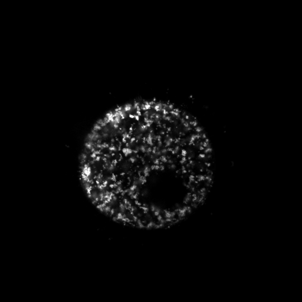

Supplement: Supplementary file 8 — Source Data [file 41467_2025_66042_MOESM8_ESM.zip › Source data/Figure 3/3h-2.tif]

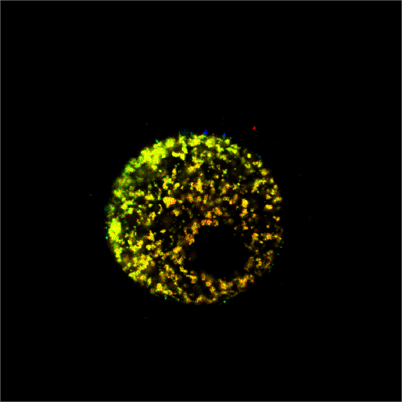

Supplement: Supplementary file 8 — Source Data [file 41467_2025_66042_MOESM8_ESM.zip › Source data/Figure 3/3h-3.tif]

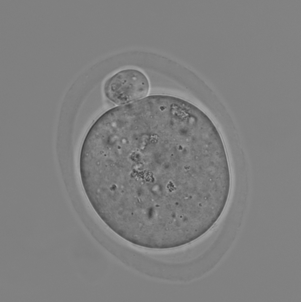

Supplement: Supplementary file 8 — Source Data [file 41467_2025_66042_MOESM8_ESM.zip › Source data/Figure 3/3h-4.tif]

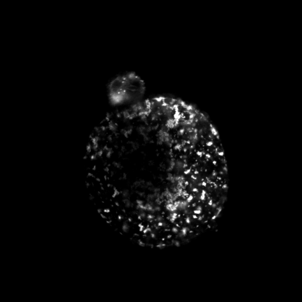

Supplement: Supplementary file 8 — Source Data [file 41467_2025_66042_MOESM8_ESM.zip › Source data/Figure 3/3h-5.tif]

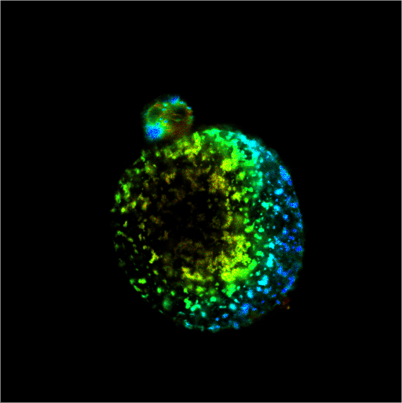

Supplement: Supplementary file 8 — Source Data [file 41467_2025_66042_MOESM8_ESM.zip › Source data/Figure 3/3h-6.tif]

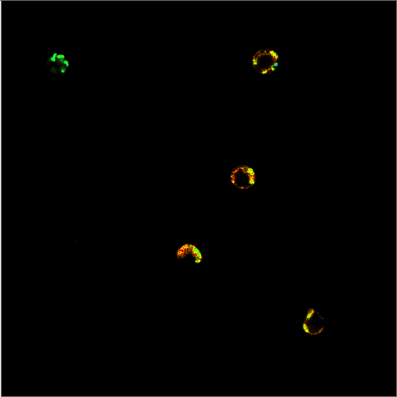

Supplement: Supplementary file 8 — Source Data [file 41467_2025_66042_MOESM8_ESM.zip › Source data/Figure 3/3j-1.tif]

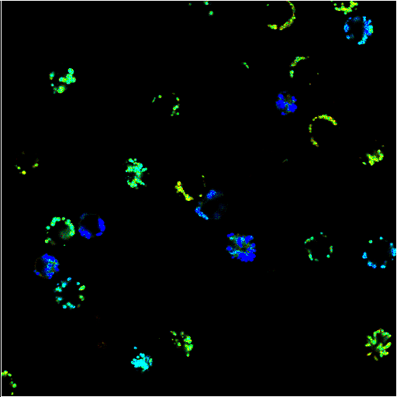

Supplement: Supplementary file 8 — Source Data [file 41467_2025_66042_MOESM8_ESM.zip › Source data/Figure 3/3j-2.tif]

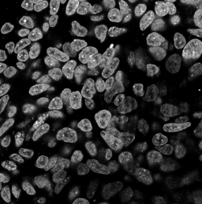

Supplement: Supplementary file 8 — Source Data [file 41467_2025_66042_MOESM8_ESM.zip › Source data/Figure 4/4a-1.tif]

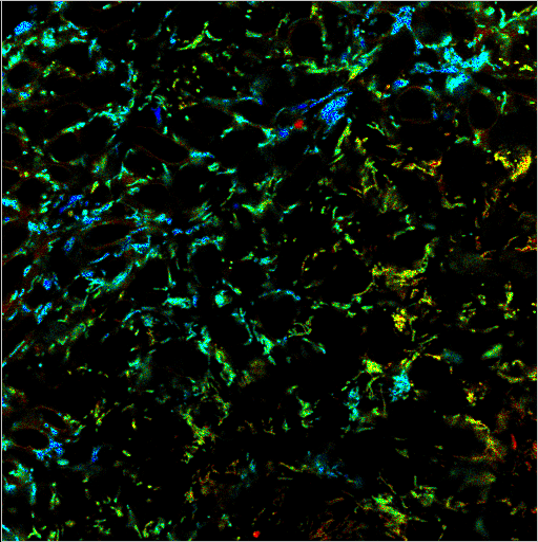

Supplement: Supplementary file 8 — Source Data [file 41467_2025_66042_MOESM8_ESM.zip › Source data/Figure 4/4a-2.tif]

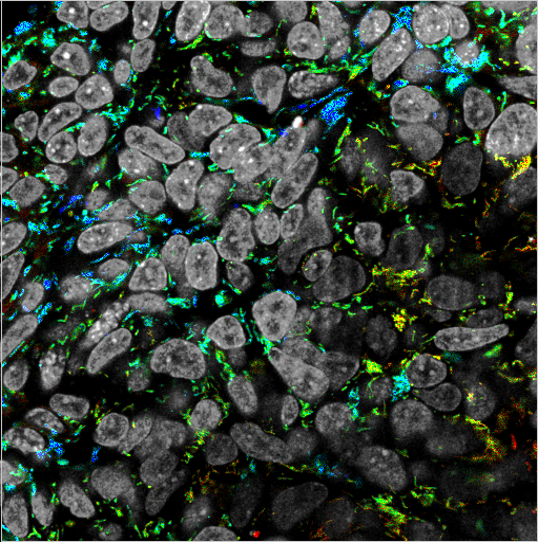

Supplement: Supplementary file 8 — Source Data [file 41467_2025_66042_MOESM8_ESM.zip › Source data/Figure 4/4a-3.tif]

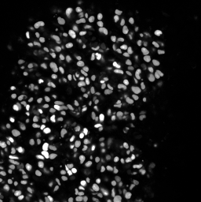

Supplement: Supplementary file 8 — Source Data [file 41467_2025_66042_MOESM8_ESM.zip › Source data/Figure 4/4a-4.tif]

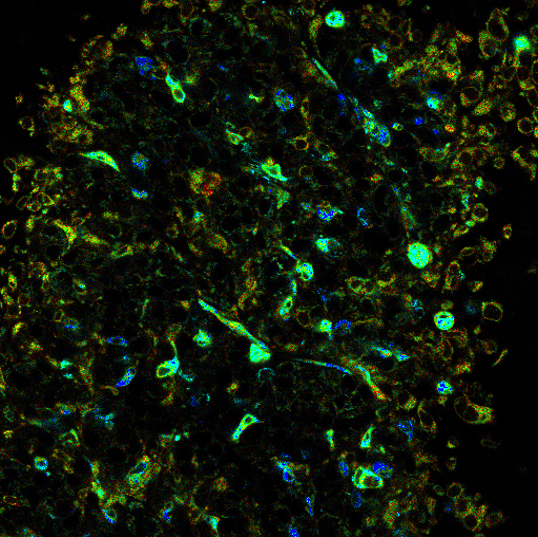

Supplement: Supplementary file 8 — Source Data [file 41467_2025_66042_MOESM8_ESM.zip › Source data/Figure 4/4a-5.tif]

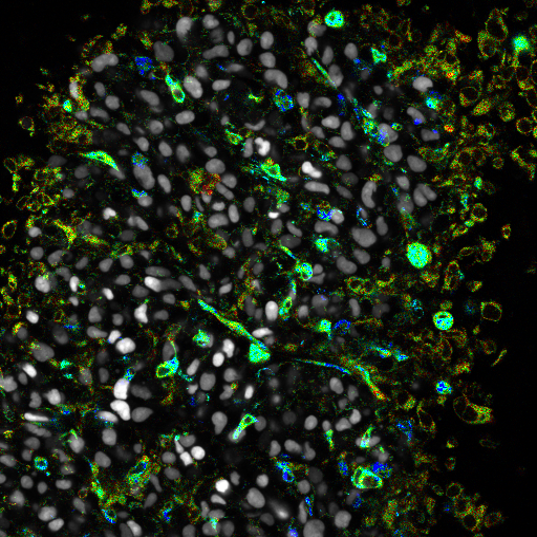

Supplement: Supplementary file 8 — Source Data [file 41467_2025_66042_MOESM8_ESM.zip › Source data/Figure 4/4a-6.tif]

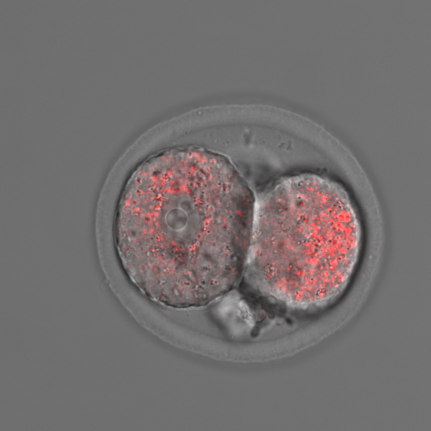

Supplement: Supplementary file 8 — Source Data [file 41467_2025_66042_MOESM8_ESM.zip › Source data/Figure 4/4b-1.tif]

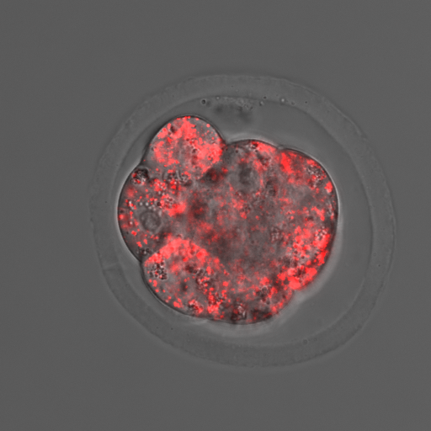

Supplement: Supplementary file 8 — Source Data [file 41467_2025_66042_MOESM8_ESM.zip › Source data/Figure 4/4b-10.tif]

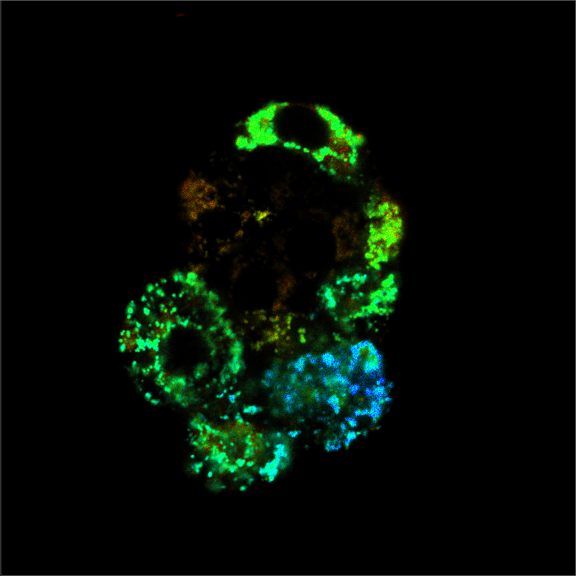

Supplement: Supplementary file 8 — Source Data [file 41467_2025_66042_MOESM8_ESM.zip › Source data/Figure 4/4b-11.tif]

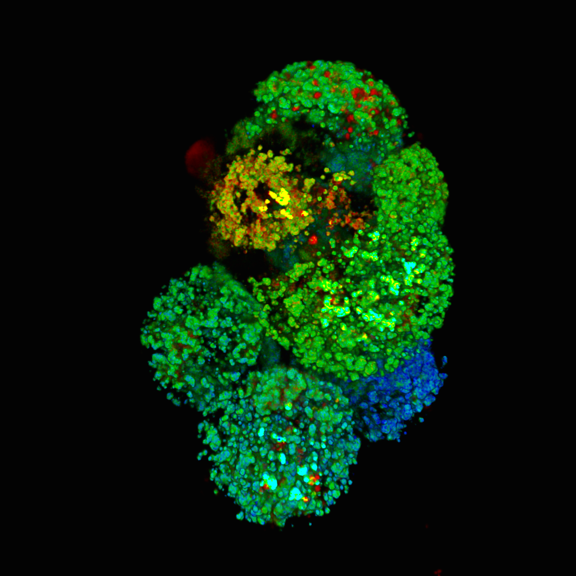

Supplement: Supplementary file 8 — Source Data [file 41467_2025_66042_MOESM8_ESM.zip › Source data/Figure 4/4b-12.tif]

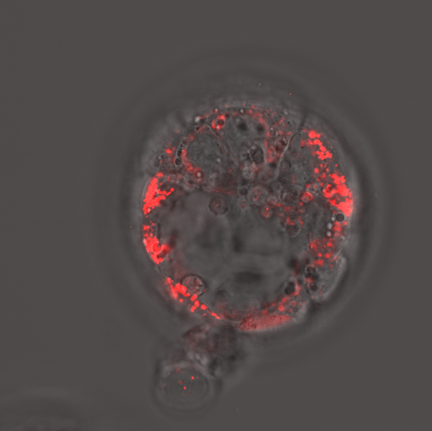

Supplement: Supplementary file 8 — Source Data [file 41467_2025_66042_MOESM8_ESM.zip › Source data/Figure 4/4b-13.tif]

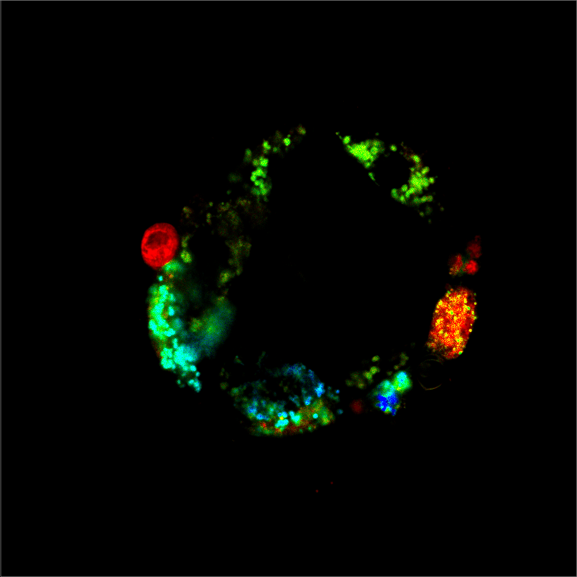

Supplement: Supplementary file 8 — Source Data [file 41467_2025_66042_MOESM8_ESM.zip › Source data/Figure 4/4b-14.tif]

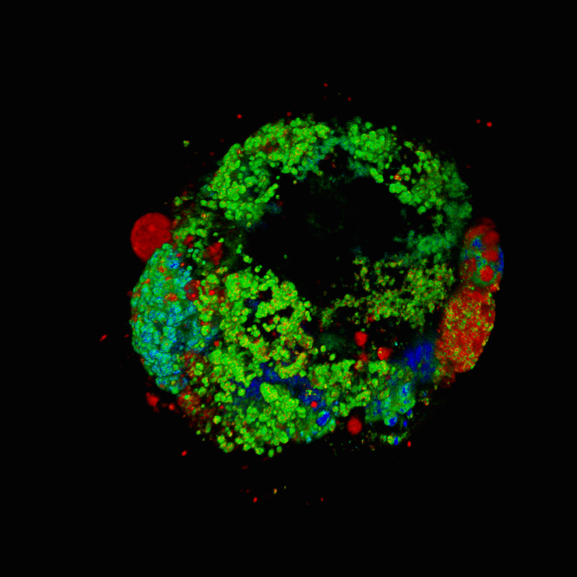

Supplement: Supplementary file 8 — Source Data [file 41467_2025_66042_MOESM8_ESM.zip › Source data/Figure 4/4b-15.tif]

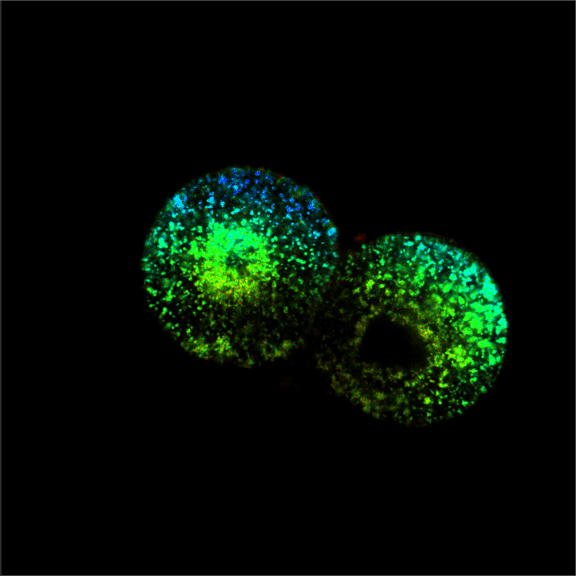

Supplement: Supplementary file 8 — Source Data [file 41467_2025_66042_MOESM8_ESM.zip › Source data/Figure 4/4b-2.tif]

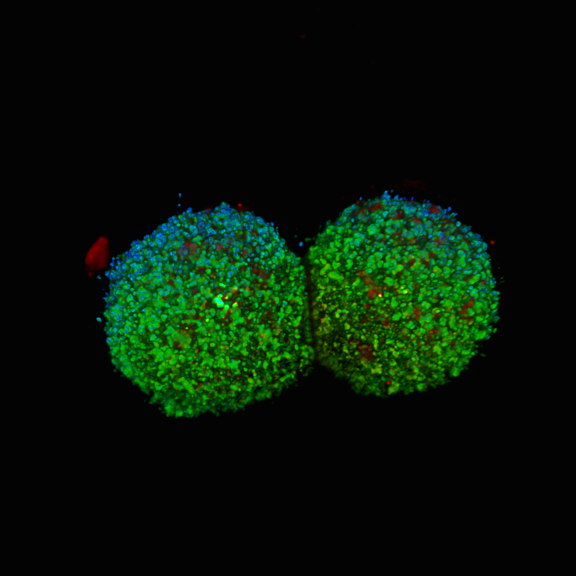

Supplement: Supplementary file 8 — Source Data [file 41467_2025_66042_MOESM8_ESM.zip › Source data/Figure 4/4b-3.tif]

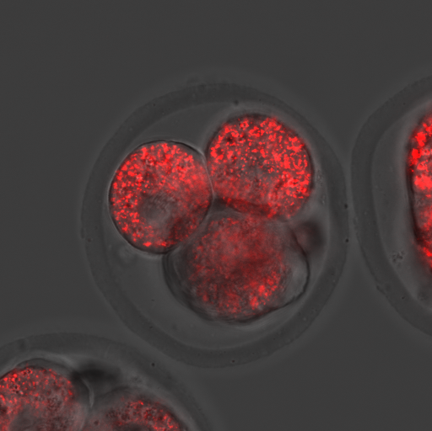

Supplement: Supplementary file 8 — Source Data [file 41467_2025_66042_MOESM8_ESM.zip › Source data/Figure 4/4b-4.tif]

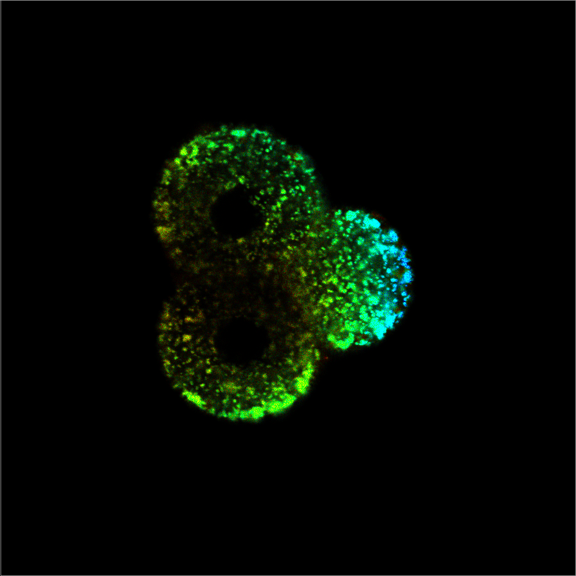

Supplement: Supplementary file 8 — Source Data [file 41467_2025_66042_MOESM8_ESM.zip › Source data/Figure 4/4b-5.tif]

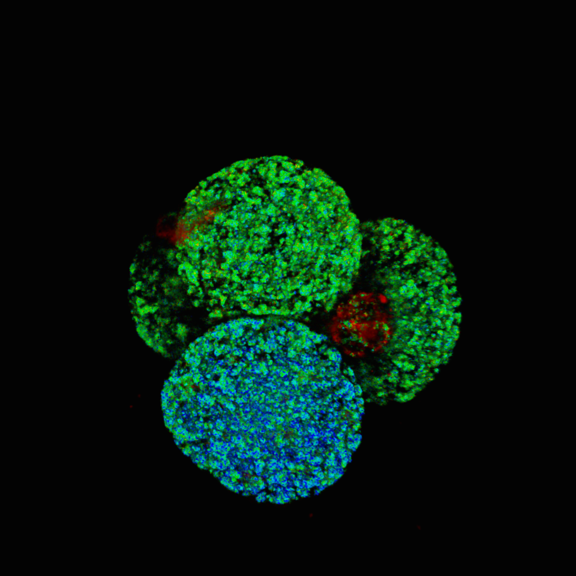

Supplement: Supplementary file 8 — Source Data [file 41467_2025_66042_MOESM8_ESM.zip › Source data/Figure 4/4b-6.tif]

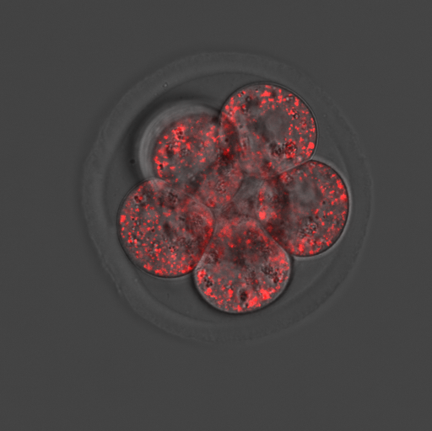

Supplement: Supplementary file 8 — Source Data [file 41467_2025_66042_MOESM8_ESM.zip › Source data/Figure 4/4b-7.tif]

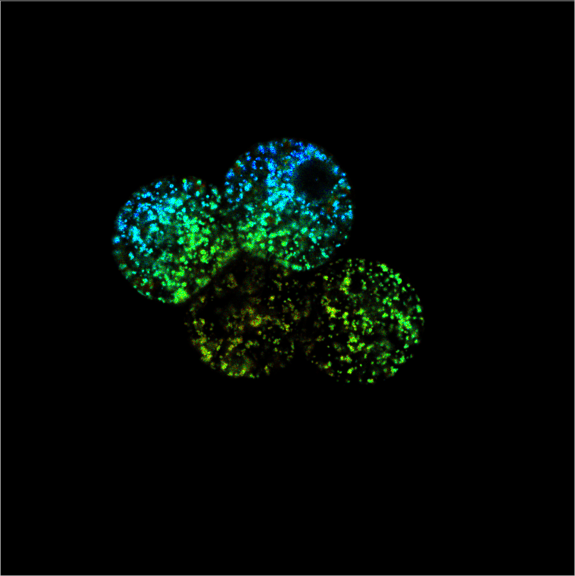

Supplement: Supplementary file 8 — Source Data [file 41467_2025_66042_MOESM8_ESM.zip › Source data/Figure 4/4b-8.tif]

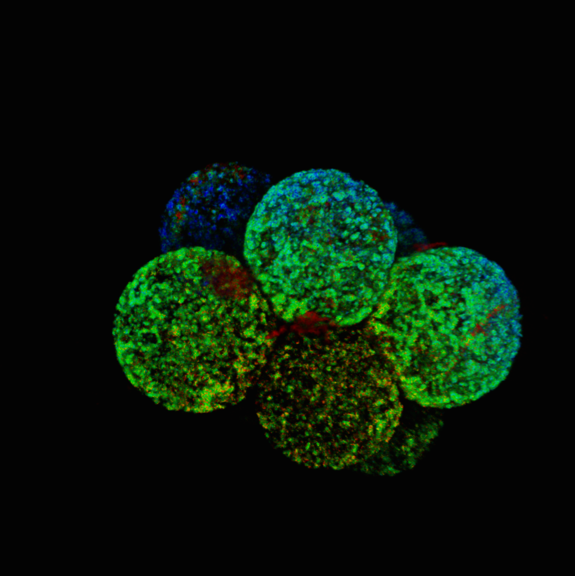

Supplement: Supplementary file 8 — Source Data [file 41467_2025_66042_MOESM8_ESM.zip › Source data/Figure 4/4b-9.tif]

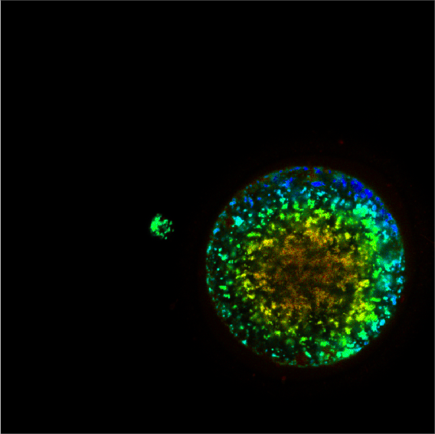

Supplement: Supplementary file 8 — Source Data [file 41467_2025_66042_MOESM8_ESM.zip › Source data/Figure 5/5a-1.tif]

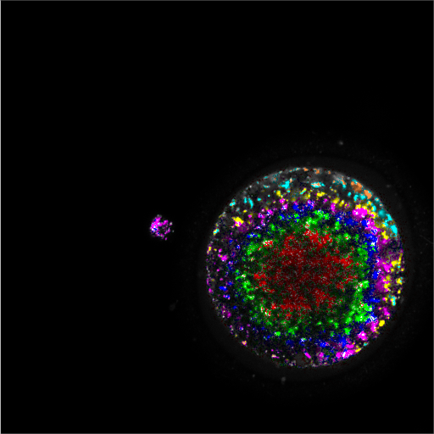

Supplement: Supplementary file 8 — Source Data [file 41467_2025_66042_MOESM8_ESM.zip › Source data/Figure 5/5a-2.tif]

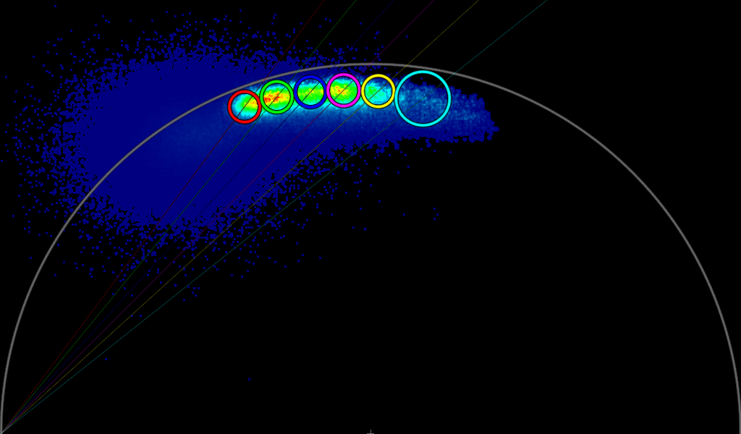

Supplement: Supplementary file 8 — Source Data [file 41467_2025_66042_MOESM8_ESM.zip › Source data/Figure 5/5a-3.tif]

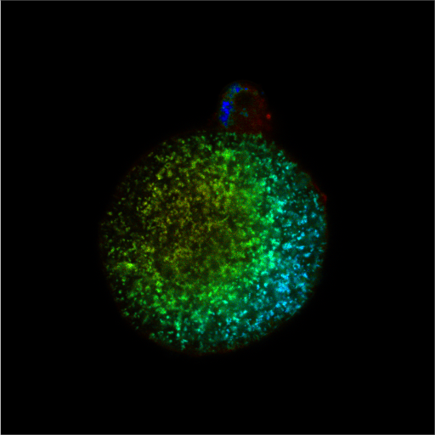

Supplement: Supplementary file 8 — Source Data [file 41467_2025_66042_MOESM8_ESM.zip › Source data/Figure 5/5b-1.tif]

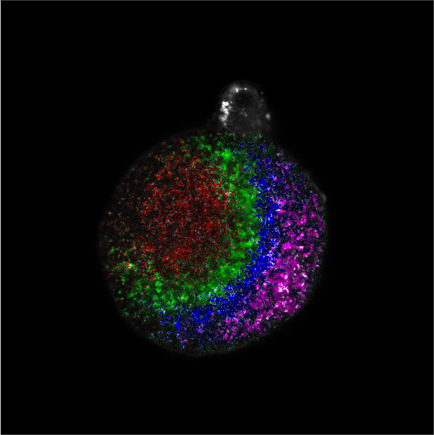

Supplement: Supplementary file 8 — Source Data [file 41467_2025_66042_MOESM8_ESM.zip › Source data/Figure 5/5b-2.tif]

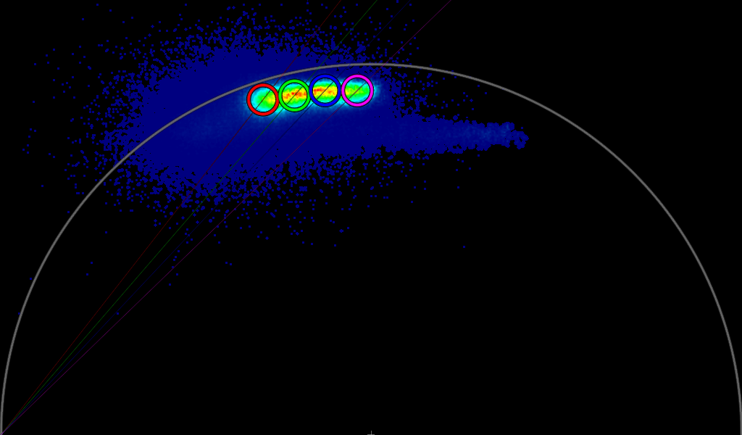

Supplement: Supplementary file 8 — Source Data [file 41467_2025_66042_MOESM8_ESM.zip › Source data/Figure 5/5b-3.tif]

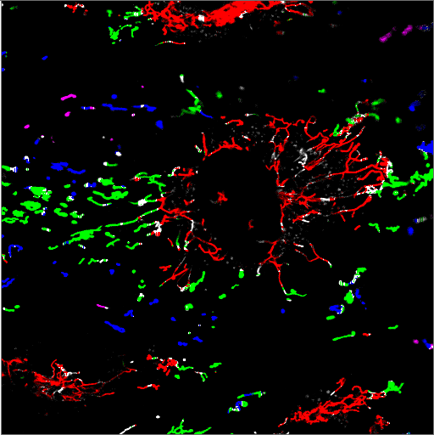

Supplement: Supplementary file 8 — Source Data [file 41467_2025_66042_MOESM8_ESM.zip › Source data/Figure 5/5c-1.tif]

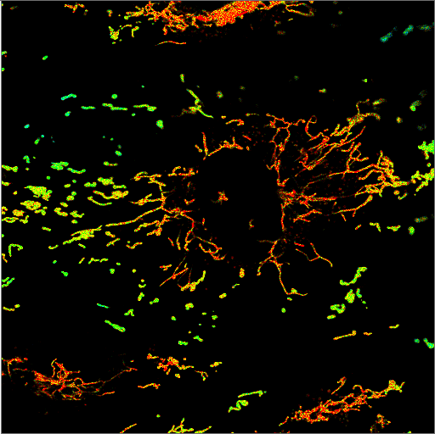

Supplement: Supplementary file 8 — Source Data [file 41467_2025_66042_MOESM8_ESM.zip › Source data/Figure 5/5c-2.tif]

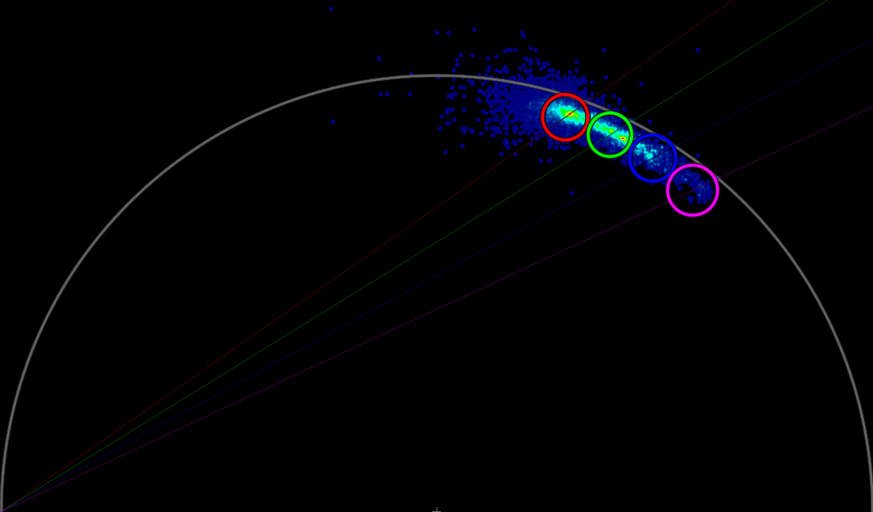

Supplement: Supplementary file 8 — Source Data [file 41467_2025_66042_MOESM8_ESM.zip › Source data/Figure 5/5c-3.tif]

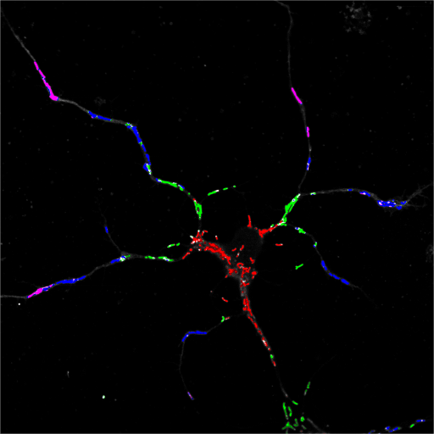

Supplement: Supplementary file 8 — Source Data [file 41467_2025_66042_MOESM8_ESM.zip › Source data/Figure 5/5d-1.tif]

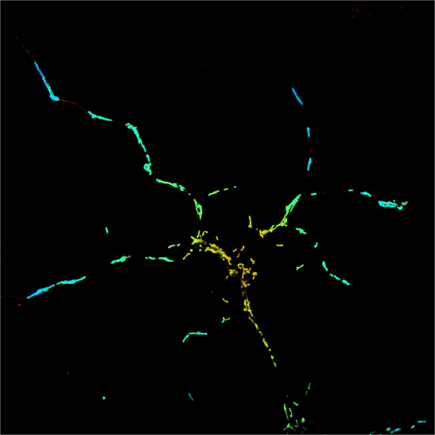

Supplement: Supplementary file 8 — Source Data [file 41467_2025_66042_MOESM8_ESM.zip › Source data/Figure 5/5d-2.tif]

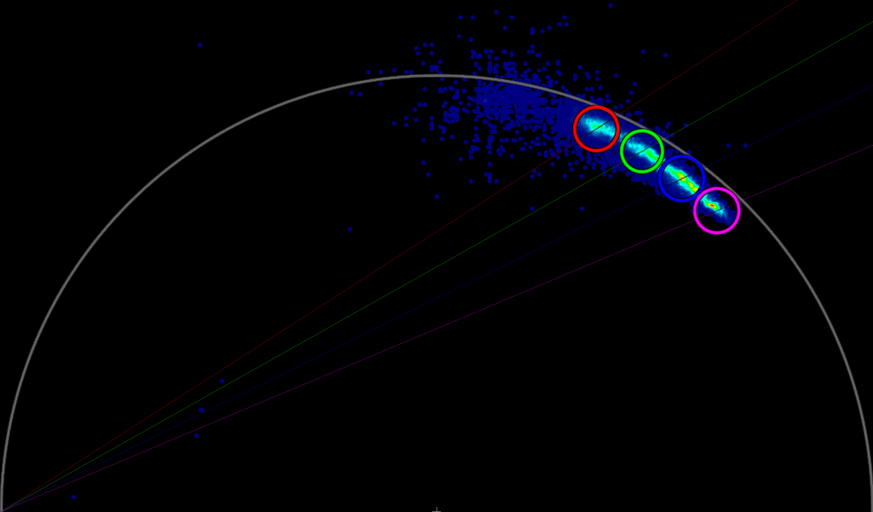

Supplement: Supplementary file 8 — Source Data [file 41467_2025_66042_MOESM8_ESM.zip › Source data/Figure 5/5d-3.tif]

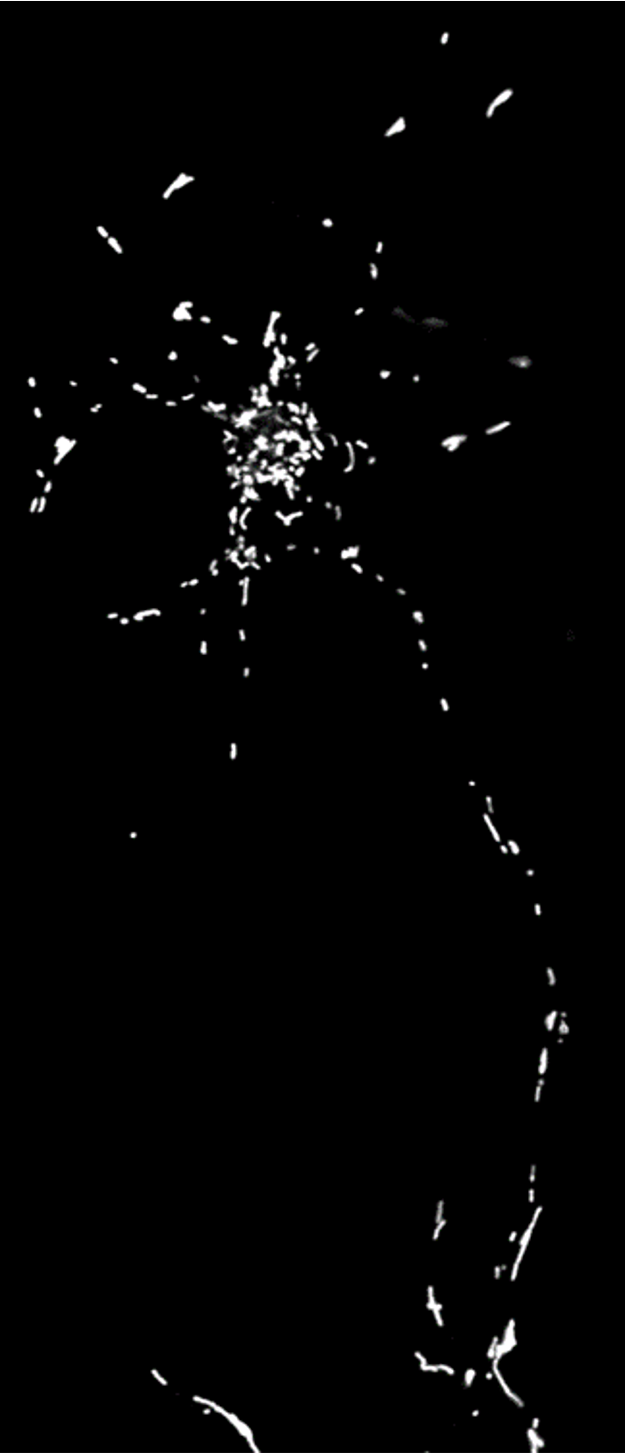

Supplement: Supplementary file 8 — Source Data [file 41467_2025_66042_MOESM8_ESM.zip › Source data/Figure 5/5e-1.tif]

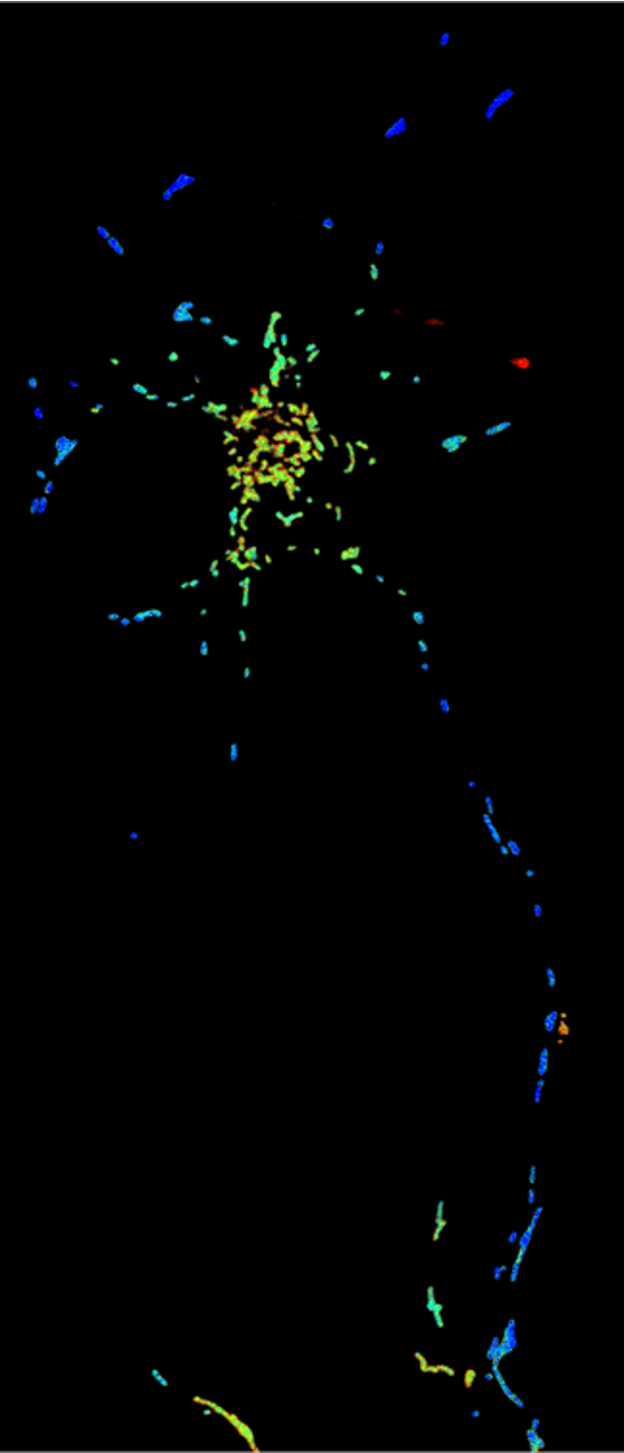

Supplement: Supplementary file 8 — Source Data [file 41467_2025_66042_MOESM8_ESM.zip › Source data/Figure 5/5e-2.tif]

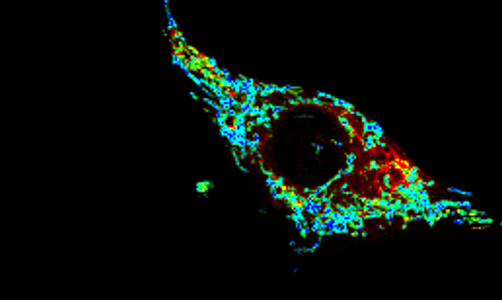

Supplement: Supplementary file 8 — Source Data [file 41467_2025_66042_MOESM8_ESM.zip › Source data/Figure 6/6a-1.tif]

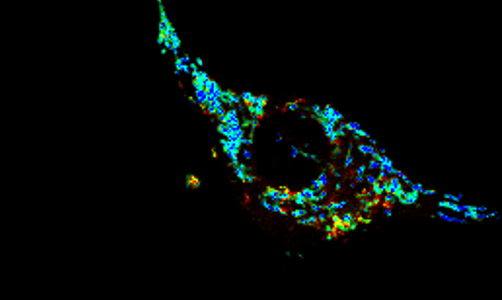

Supplement: Supplementary file 8 — Source Data [file 41467_2025_66042_MOESM8_ESM.zip › Source data/Figure 6/6a-10.tif]

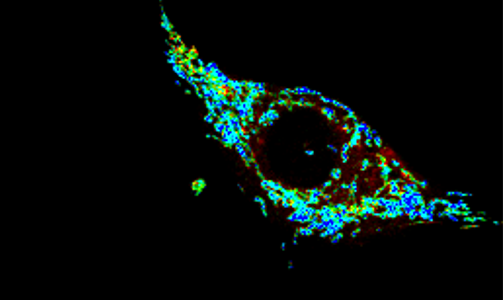

Supplement: Supplementary file 8 — Source Data [file 41467_2025_66042_MOESM8_ESM.zip › Source data/Figure 6/6a-2.tif]

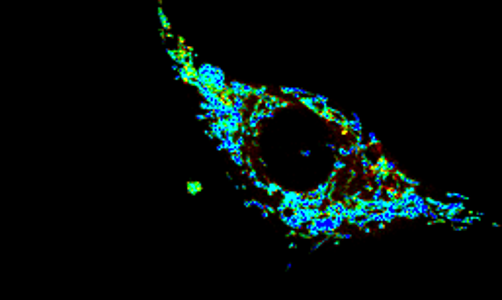

Supplement: Supplementary file 8 — Source Data [file 41467_2025_66042_MOESM8_ESM.zip › Source data/Figure 6/6a-3.tif]

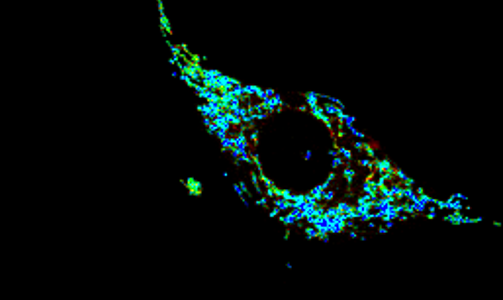

Supplement: Supplementary file 8 — Source Data [file 41467_2025_66042_MOESM8_ESM.zip › Source data/Figure 6/6a-4.tif]

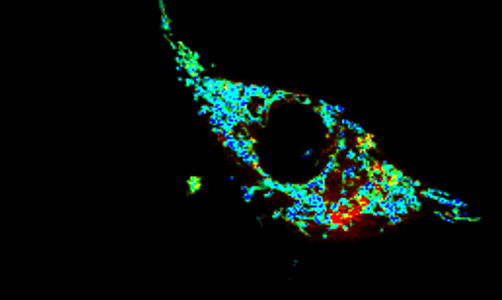

Supplement: Supplementary file 8 — Source Data [file 41467_2025_66042_MOESM8_ESM.zip › Source data/Figure 6/6a-5.tif]

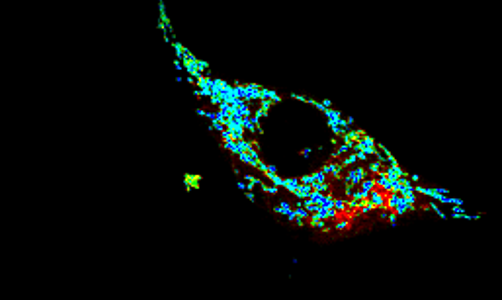

Supplement: Supplementary file 8 — Source Data [file 41467_2025_66042_MOESM8_ESM.zip › Source data/Figure 6/6a-6.tif]

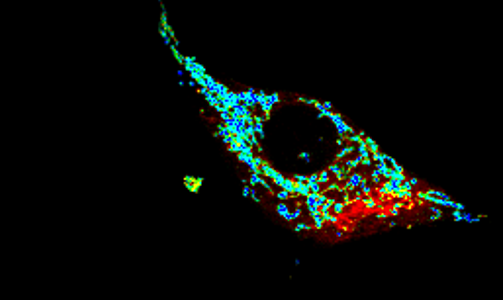

Supplement: Supplementary file 8 — Source Data [file 41467_2025_66042_MOESM8_ESM.zip › Source data/Figure 6/6a-7.tif]

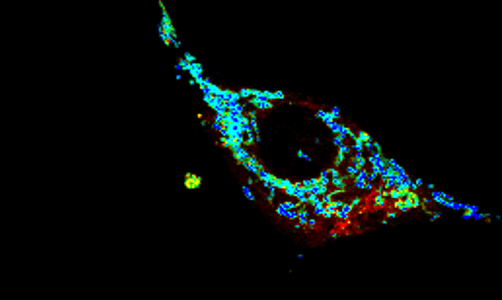

Supplement: Supplementary file 8 — Source Data [file 41467_2025_66042_MOESM8_ESM.zip › Source data/Figure 6/6a-8.tif]

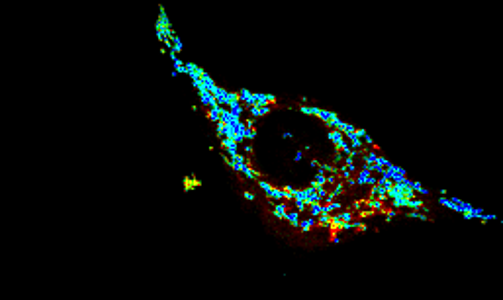

Supplement: Supplementary file 8 — Source Data [file 41467_2025_66042_MOESM8_ESM.zip › Source data/Figure 6/6a-9.tif]

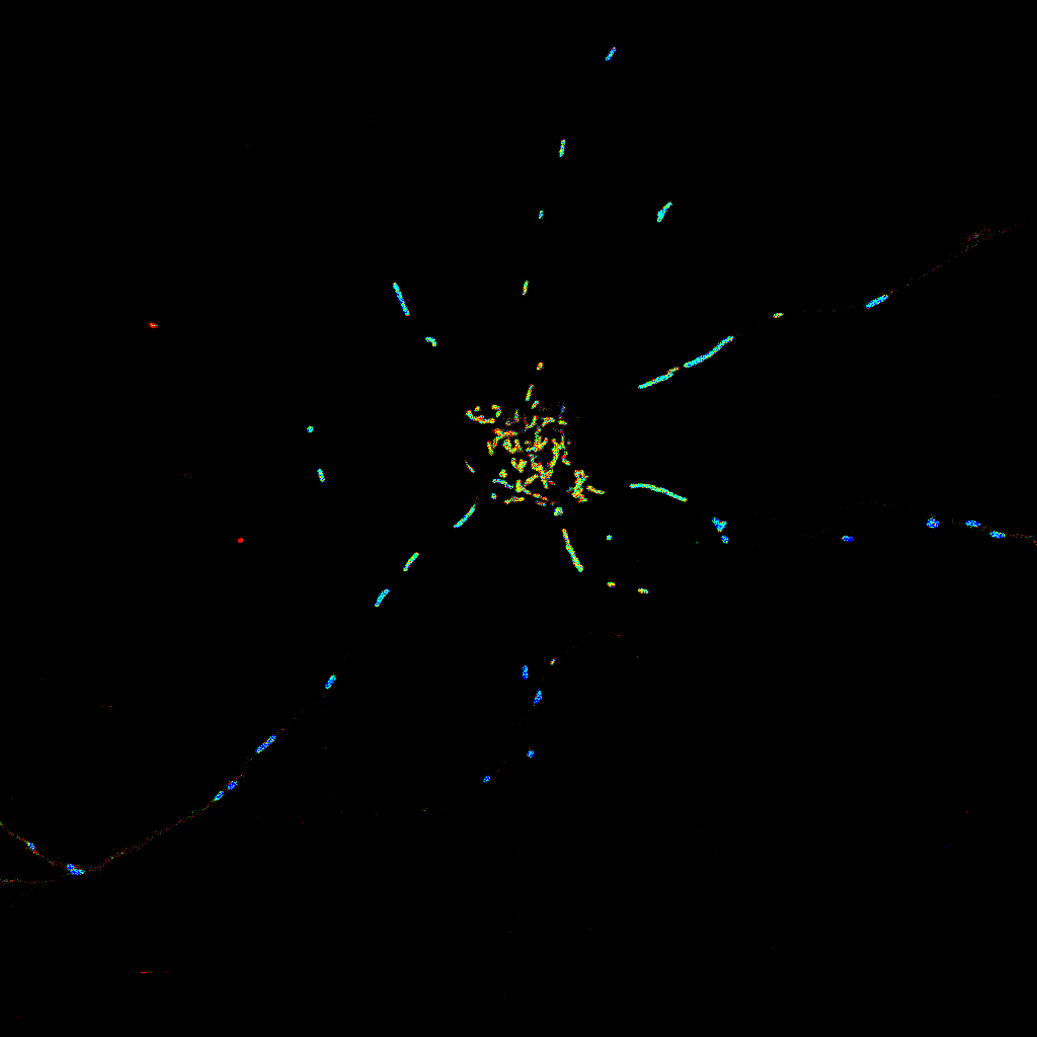

Supplement: Supplementary file 8 — Source Data [file 41467_2025_66042_MOESM8_ESM.zip › Source data/Figure 6/6c-1.tif]

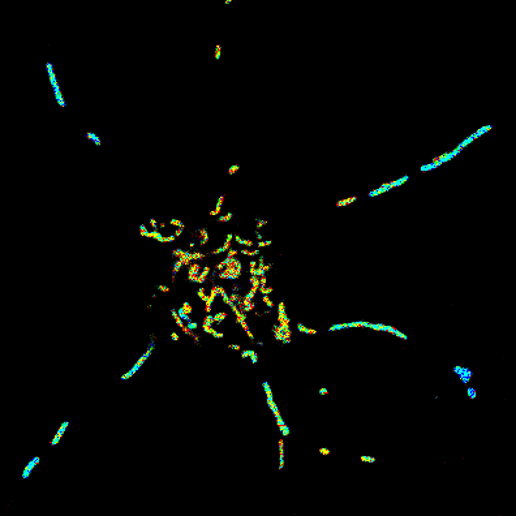

Supplement: Supplementary file 8 — Source Data [file 41467_2025_66042_MOESM8_ESM.zip › Source data/Figure 6/6c-2.tif]
